# Analysis of COVID-19 clinical trials: A data-driven, ontology-based, and natural language processing approach

**DOI:** 10.1371/journal.pone.0239694

**Published:** 2020-09-30

**Authors:** Shray Alag

**Affiliations:** The Harker School, San Jose, CA, United States of America; National Library of Medicine, UNITED STATES

## Abstract

With the novel COVID-19 pandemic disrupting and threatening the lives of millions, researchers and clinicians have been recently conducting clinical trials at an unprecedented rate to learn more about the virus and potential drugs/treatments/vaccines to treat its infection. As a result of the influx of clinical trials, researchers, clinicians, and the lay public, now more than ever, face a significant challenge in keeping up-to-date with the rapid rate of discoveries and advances. To remedy this problem, this research mined the ClinicalTrials.gov corpus to extract COVID-19 related clinical trials, produce unique reports to summarize findings and make the meta-data available via Application Programming Interfaces (APIs). Unique reports were created for each drug/intervention, Medical Subject Heading (MeSH) term, and Human Phenotype Ontology (HPO) term. These reports, which have been run over multiple time points, along with APIs to access meta-data, are freely available at http://covidresearchtrials.com. The pipeline, reports, association of COVID-19 clinical trials with MeSH and HPO terms, insights, public repository, APIs, and correlations produced are all novel in this work. The freely available, novel resources present up-to-date relevant biological information and insights in a robust, accessible manner, illustrating their invaluable potential to aid researchers overcome COVID-19 and save hundreds of thousands of lives.

## Introduction

Since the onset of the Coronavirus disease 2019 (COVID-19) pandemic, researchers and clinicians have been swiftly conducting clinical trials to better understand the virus, its transmission, and potential drugs and vaccines to counter its rapid spread. Such COVID-19 related clinical trials can be found at ClinicalTrials.gov, a database for globally-conducted clinical trials run by the United States National Library of Medicine.

The number of COVID-19 related clinical trials is dramatically increasing: There were approximately 500 clinical trials in mid-late April, more than 1000 in early May, over 2000 in early June, and over 3000 in mid-July [1]. By late August, this number has increased to nearly 3,500 clinical trials. As a result of the unprecedented volume of new clinical trials, the task of staying informed about crucial developments and ongoing research is not only arduous but also extremely time-consuming. Ignorance of recent advancements could result in misconceptions, the misuse of time, and non-optimal allocation of funds/resources. Therefore, improved accessibility of information about COVID-19 related clinical trials would aid clinicians, researchers, and the lay public alike.

This research followed seven principal steps:

1. Obtain all COVID-19 related clinical trials from ClincialTrials.gov.
2. Extract relevant Interventions, Drugs, Outcomes, Location, Medical Subject Heading (MeSH [2]) tags, and more information from the identified clinical trials.
3. Associate the clinical trial with a Human Phenotype Ontology (HPO [3]) term, if applicable, through using the MeSH tags.
4. Correlate Drugs, MeSH terms, and HPO terms computationally by examining the frequency of these elements in COVID-19 clinical trials. In essence, for a given term of interest compute other correlated terms [4, 5].
5. Generate unique publicly-accessible, informative—yet concise—reports for each of the Intervention/Drug terms, MeSH terms, and HPO terms.
6. Create a freely-available public repository detailing associations between Interventions/Drugs, MeSH terms, and HPO terms while additionally providing access to Application Programming Interfaces (APIs), which enable the user to interact with the data using a programming language, such as Java or Python.
7. Analyze the clinical trials at multiple time points, enabling future meta-analyses.

## Materials and methods

This work builds upon the pipeline developed in Alag 2020 [6]. The core of the pipeline and methodology is publicly available at protocols.io (dx.doi.org/10.17504/protocols.io.bfacjiaw). As detailed in the protocols, the following online repositories/vocabularies were used:

1. ClinicalTrials.gov [7]: The complete repository of clinical trials displayed at ClinicalTrials.gov is available in extensible Markup Language (XML) format with a well-defined schema [8].
2. Human Phenotype Ontology (HPO [3]): HPO is a standardized vocabulary of phenotype abnormalities that are seen in humans [3].
3. Medical Subject Headings (MeSH [2]): The ClinicalTrials.gov XML contains information about MeSH terms, and, using the MeSH online tool [9], MeSH ids were retrieved from their corresponding MeSH terms.

The predominant differences between the methodology employed here and that followed in Alag 2020 [6] are detailed in the following subsections. Readers are encouraged to refer to Alag 2020 for more in-depth details. It is relevant to note that MeSH and HPO ontologies were chosen over multiple alternative ontologies as these were easily compatible with the clinical trails XML format, enabling higher-level correlations across related genes, SNPs, protein mutations, and even clinical trials. Additionally, the additional feature of correlating COVID-19 clinical trials to SNPs and protein mutations is provided through utilizing the HPO ontology and previous work in Alag 2020 [6].

### Identifying COVID-19 clinical trials

To accurately and efficiently identify COVID-19 trials, sections of the text of the trial—the title, brief summary, outcomes sections, clinical trial criteria, conditions, MeSH terms, and detailed description—were seen to have a case-insensitive match to any of the following terms: COVID 19, COVID-19, SARS-CoV-2, 2019-ncov, coronavirus, severe acute respiratory syndrome coronavirus 2, 2019 novel coronavirus, and wuhan coronavirus. These terms were selected after reviewing various sites, including clinicaltrials.gov [10], that have kept a running list of COVID-19-related publications and clinical trials. The research attempts to ensure no COVID-19 clinical trials are overlooked, and, as a result, the terms list is more extensive than the parameters clinicaltrials.gov uses in its COVID-19 search. These terms were kept constant to be able to analyze longitudinal trends without bias.

### Analysis pipeline

To analyze the clinical trials, a methodology similar to that of Alag 2020 [6] was used with the following additions. It is important to note that interventions are the focus of a clinical trial: often in a clinical trial, the response of patients who are given an intervention (drug, test, procedure, etc.) are compared to patients who do not receive that intervention. Interventions can be drugs, medical devices, vaccines, procedures, genetic tests, noninvasive techniques, such as diet, education, or exercise, and are sorted into eleven different categories (e.g., genetic, radiation, etc.).

1. Interventions/Drugs: A list of unique interventions/drugs that appear in COVID-19 related clinical trials were created. This dictionary of terms was essential to later formulate reports and correlations. The data was retrieved at multiple time points to get insights about the rate at which these trials are occurring.
2. Correlations: Through using co-occurrences of a specific term, correlations were noted between drugs/interventions, MeSH terms, and HPO terms, as further described in the following subsection.
3. Reports: Unique hypertext markup language (HTML)-based reports were created for each of the interventions/drugs, MeSH tags, and HPO terms. These reports have associated clinical trials and related HPO terms, each of which also has associated genes, SNPs, and protein mutations. A more in-depth discussion of the reports is provided in the Results section.

### Computing correlations

The co-occurrence of a term across different clinical trials was used to compute correlations between terms using the following procedure [4]:

1. Create an incidence matrix where each row is a term of interest (drug, MeSH, or HPO). By doing so, there are *m* such terms and *n* clinical trials. A value of one is marked each time the *m*_*i*_ term is correlated with the *n*_*j*_ clinical trial. All other non-correlated positions should have a value of zero.
2. Normalize the data by creating a unit vector for each term. Unit vectors are obtained by dividing each element of a row by the magnitude of that row.
3. For each term, compute the pair-wise dot product between its vector and all other vectors. The resulting number is a measure of normalized correlation.
4. Sort the results to create a prioritized list of related terms.

Hierarchical Clustering [11] or K-Means [12] could also be used to find clusters of related terms. Additionally, the rows and columns can be switched to cluster similar clinical trials by their associated terms [5].

## Results

The “Results” section is comprised of discussions about the following three main areas:

1. Details on the created public repository to provide access to the data used, reports created, correlations mapped, and APIs produced.
2. Insights from analyzing COVID-19 clinical trials.
3. Findings related to correlations.

### Public repository

#### Web page to access longitudinal analysis data, reports, and APIs

All analysis results are accessible via the Covid Research Trials home page, available at http://CovidResearchTrials.com. A view of the home page is seen in S1 File. The web page provides access to data and reports from multiple time frames: At the date of publication, information from May 2nd, 2020, May 23rd, 2020, June 6th, July 18th, and August 16th, 2020 are illustrated. Although subject to change, the Covid Research Trials home page provides the latest analysis results. Additionally, the home page has links to numerous Java APIs and a Google Colab page, which facilitate easy local access to this research’s insights and results. The functionalities of the various APIs are to retrieve information about the following:

1. A list of all COVID-19 related clinical trials along with the description of each trial
2. Potential vaccines and their corresponding clinical trials
3. Drugs and their corresponding clinical trials
4. COVID-19 related HPO terms and their correlated clinical trials
5. COVID-19 related MeSH terms along with their corresponding clinical trials
6. COVID-19 related clinical trials and the outcomes of each of the trials

Each Java class is a stand-alone program and does not require any other package beyond the Java core classes: Users can simply download a Java IDE, install Java, and run the class on that IDE. S2 File contains screenshots of the documentation of each of the six APIs mentioned above. The main function in each of the classes demonstrates how each of the public methods can be called. Additionally, the Google Colab Notebook, which uses Python, reads in and details information about all relevant clinical trials, the tested drugs, and the potential vaccines.

### Insights from analyzing COVID-19 clinical trials

The following subsections detail trends gleaned from analyzing the longitudinal data, meta-level information about COVID-19 related clinical trials, key intervention/drug, MeSH, and HPO terms, and provide information about the generated reports.

#### Longitudinal information associated with COVID-19

**Fig 1:**
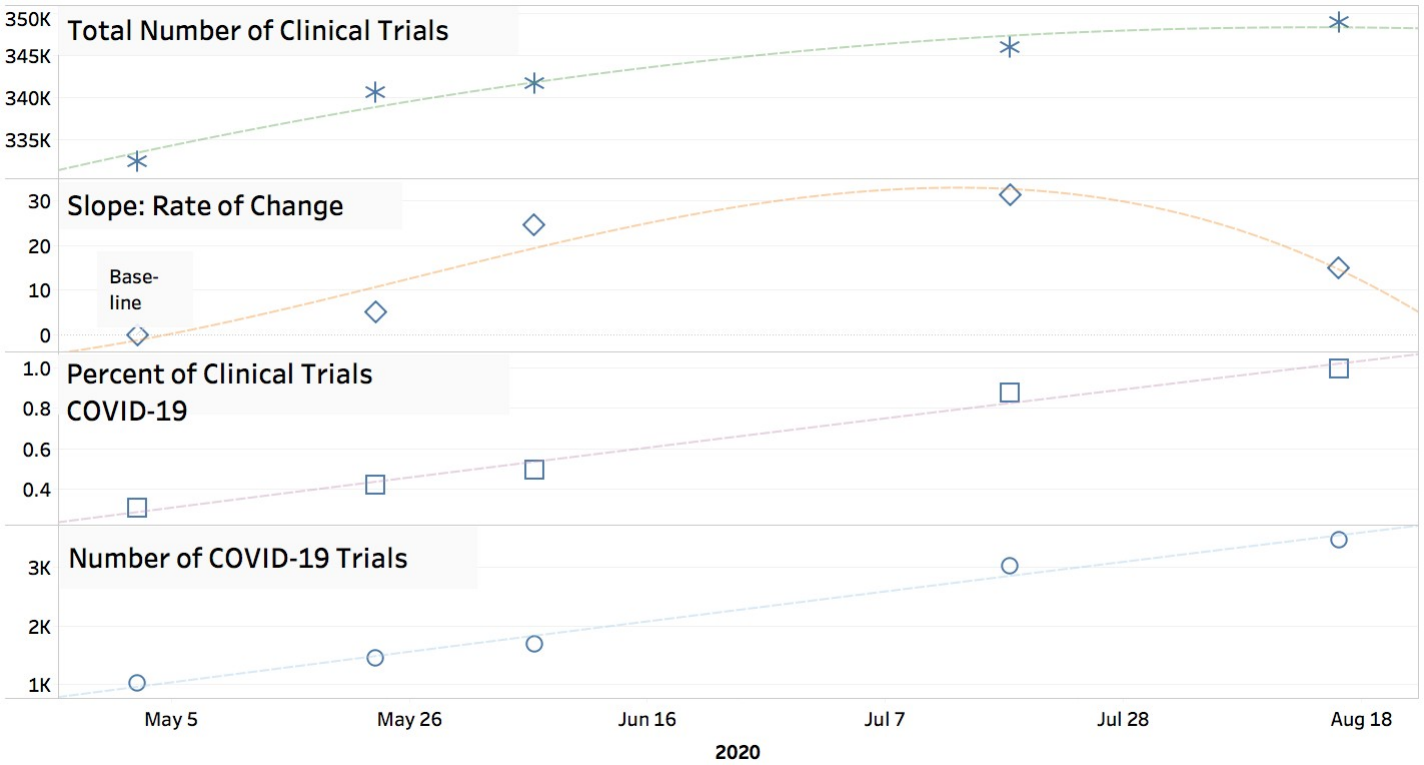
COVID-19 clinical trial trends: Longitudinal trends from COVID-19 related clinical trials. The data is plotted across five time points: May, 3rd 2020, May, 23rd, 2020, June 6th, 2020, July 18th, 2020, and August 16, 2020. (a) The total number of clinical trials at each time points; (b) Percent of new clinical trials (trials published between time segments) that are COVID-19 related; (c) Percent of all clinical trials that are COVID-19 related; (d) The total number of COVID-19 related clinical trials.

Fig 1 illustrates the trends of COVID-19 clinical trials, with the key takeaways being the following:

1. The number of COVID related trials has been linearly increasing with approximately 120 new clinical trials per week. As of late August 2020 there were nearly 3,500 COVID-19 related clinical trials.
2. Out of the more recent trials, an increasing percentage of all trials are COVID-19 related: in the first period approximately 5% of all new trials were COVID related while around a quarter of new clinical trials from May 23rd to June 6th are COVID-19 related. In July-August 2020, the rate of change of new COVID-19 clinical trials appears to be slowing.
3. The percent of COVID-19 clinical trials, as a percentage of all clinical trials, is close to 1% as of late August 2020.

The methodology employed in this research will continue to be performed at future time points, and the future changes in trends can provide interesting insights.

#### Key interventions/drugs associated with COVID-19

**Fig 2:**
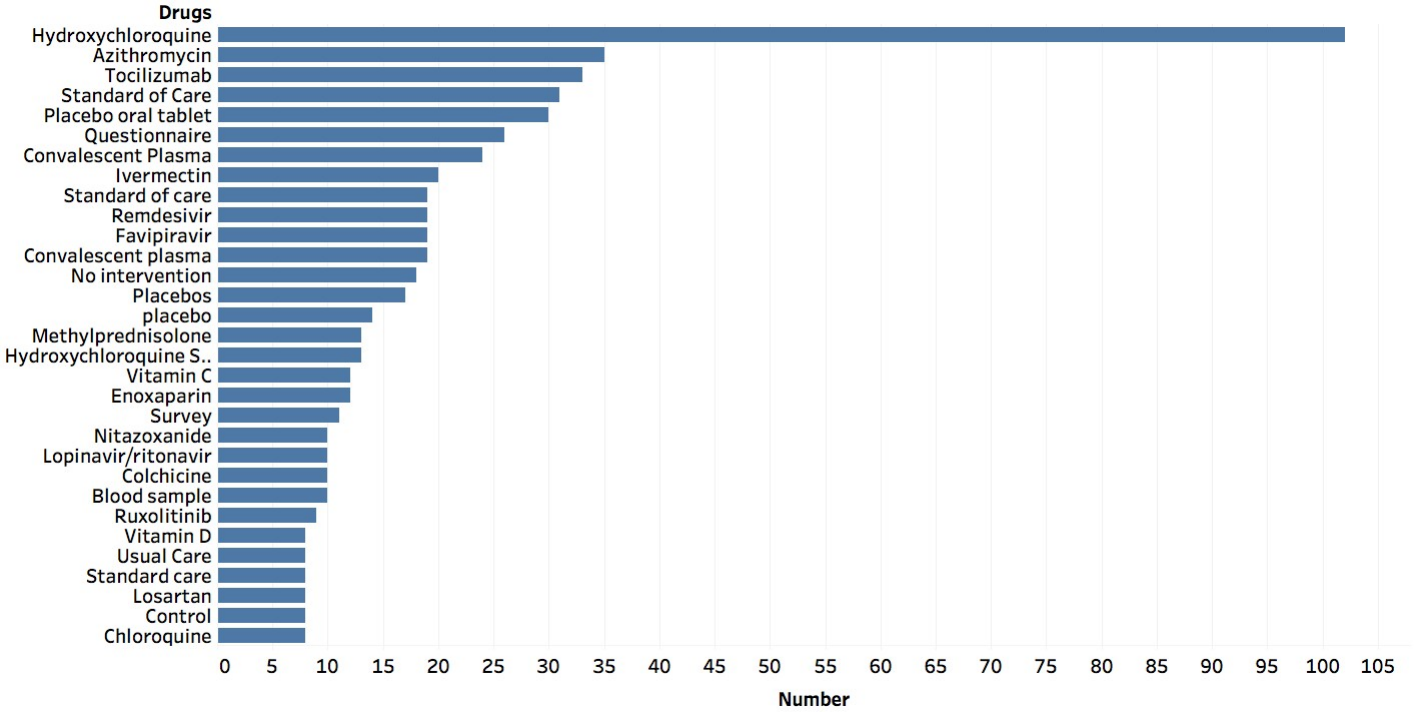
Intervention/Drug information. The graph shows the relative frequency of the different intervention/drugs that are referenced in COVID-19 related clinical trials (August 2020). A minimum occurrence of 8 clinical trials was used to create the graph.

At the last data point, on August 16th, 2020, there were 3,523 unique interventions/drugs that were associated with COVID-19 clinical trials. Fig 2 shows the most-frequently occurring interventions/drugs for COVID-19 related clinical trials. The most popular interventions being tested are Hydroxychloroquine, Azithromycin, Tocilizumab, Standard of Care, Placebo, Convalescent Plasma, Ivermectin, and Remdesivir, as seen in Fig 2. Additionally, it is important to note that Hydroxychloroquine, with 102 trials, is the most common drug that is being tested in clinical trials.

**Fig 3:**
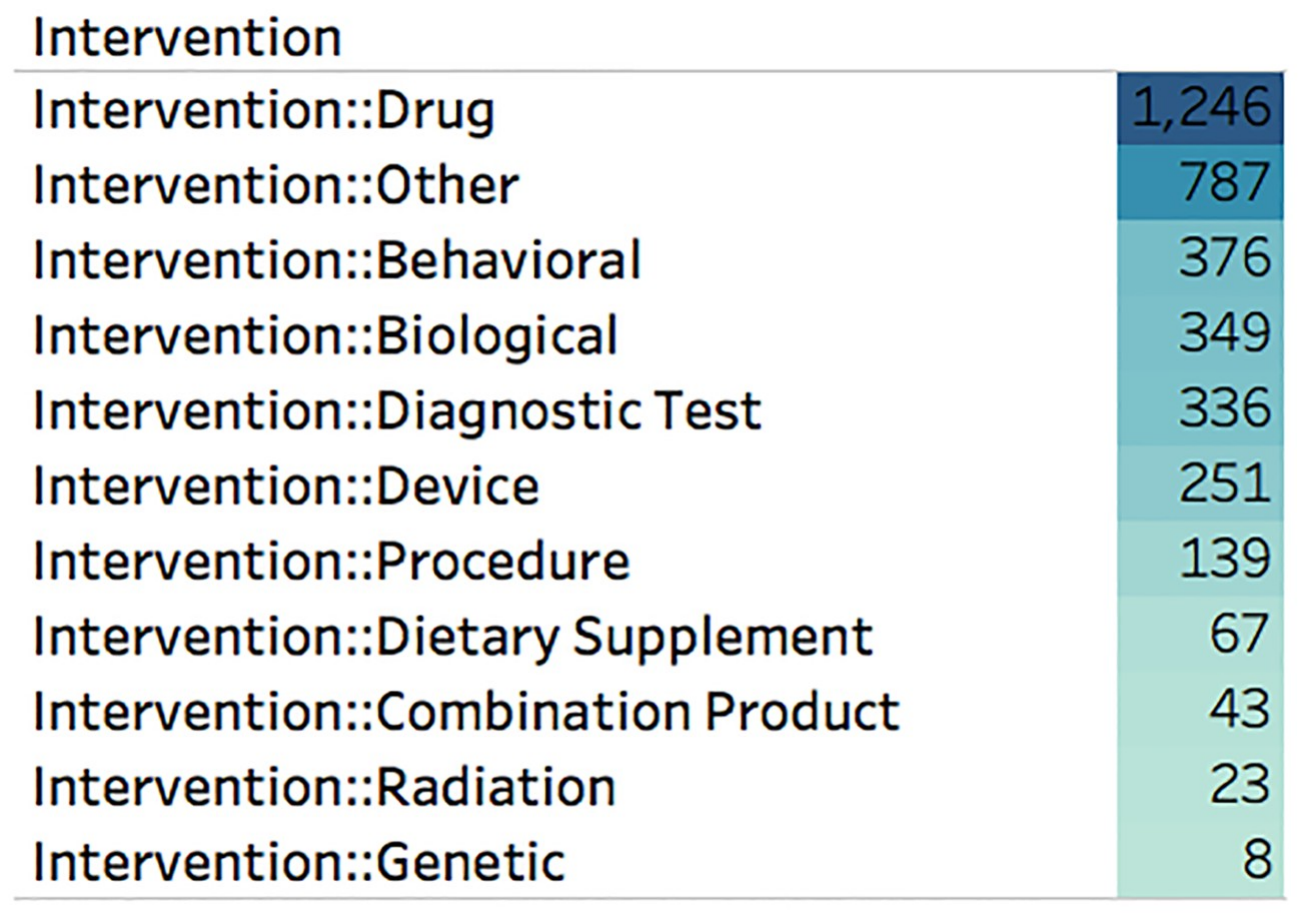
Intervention-categories. The majority of the interventions used in clinical trials are drugs, other, behavioral, biological, and diagnostic tests (August 2020).

Fig 3 depicts the frequency of intervention types across COVID-19 related trials, illustrating that the most popular intervention category is Drug, followed by Other, Behavioral, Biological, and Diagnostic test. As most COVID-19 clinical trials are either experimenting with the efficacy of drugs, observing behavioral changes, creating/validating diagnostic tests, or discovering the biological effects of the virus, the high intervention frequency occurrences of drug, behavioral, biological, and diagnostic test validate the procedure employed. It is interesting to see the emergence of clinical trials associated with genetic information (though just eight), and future changes in the frequencies of interventions will be insightful, especially as researchers begin looking to better understand the role of genomic factors in symptoms and recovery of patients with COVID-19.

#### Outcomes, phase, and status of COVID-19 clinical trials

**Fig 4:**
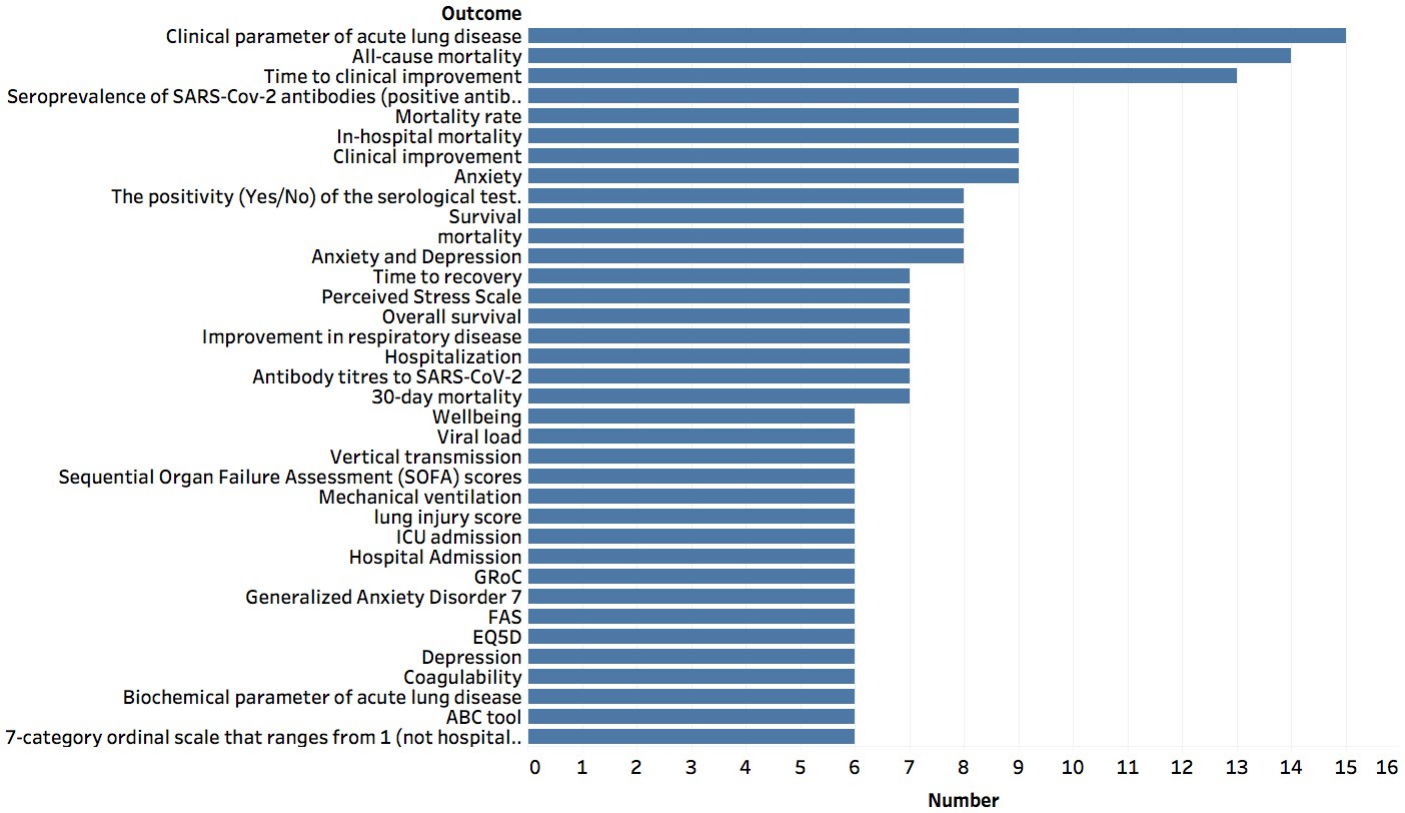
Outcomes are events that are being monitored in a clinical trial. This graph shows the most commonly occurring outcomes that are being measured in COVID-19 related clinical trials. Here a threshold of six was used to list the outcome.

**Fig 5:**
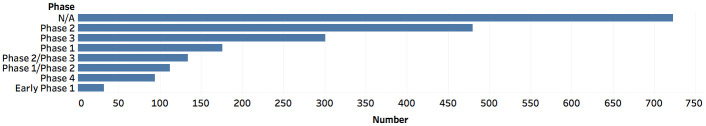
Phase information including phase 0, the FDA defines a five phase process for approving new drugs [14]. This graph shows the phases for COVID-19 related clinical trials.

**Fig 6:**
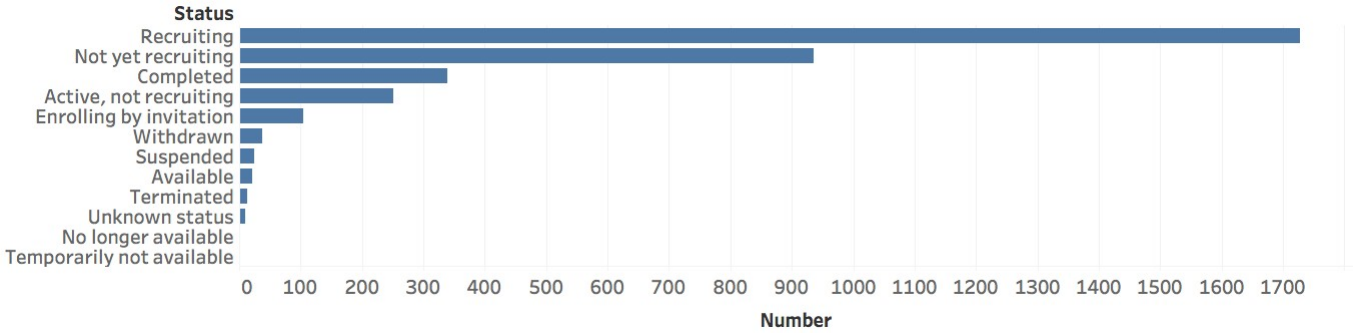
Recruitment status indicates where the clinical trial is with respect to recruiting new patients. This graph shows the number of clinical trials in each status state.

Analyzing the COVID-19 related clinical trials provides the following insights:

1. Outcomes are events (e.g., patient death or discharge from the hospital) that are actively being monitored in a clinical trial [13]. As depicted in Fig 4, the majority of outcomes either deal with acute lung disease, time to clinical improvement, antibodies, or, unfortunately, mortality.
2. Including Phase 0, the Food and Drug Administration (FDA [14]) defines a five-phase [15] approach for ensuring the safety and efficacy of an intervention. Fig 5 illustrates that the majority of the COVID-19 related clinical trials are in Phases 2 and 3, indicating that they have made considerable headway but are still far from completion. Notably, there are many very early phase trials indicating the recent interest in performing COVID-19 related studies. Encouragingly, there are quite a few clinical trials that are in Phase 4.
3. Recruitment status [16] indicates the degree to which a trial may need to enroll subjects. Fig 6 shows that the vast majority of COVID-19 clinical trials are either still recruiting participants or have not yet begun recruiting, illustrating that most of these trials are still far from complete. Further, the preliminary stages of these clinical trials indicates that they have been recently undertaken, portraying the growing interest in COVID-19 clinical trials.

#### MeSH and HPO terms associated with COVID-19 clinical trials

**Fig 7:**
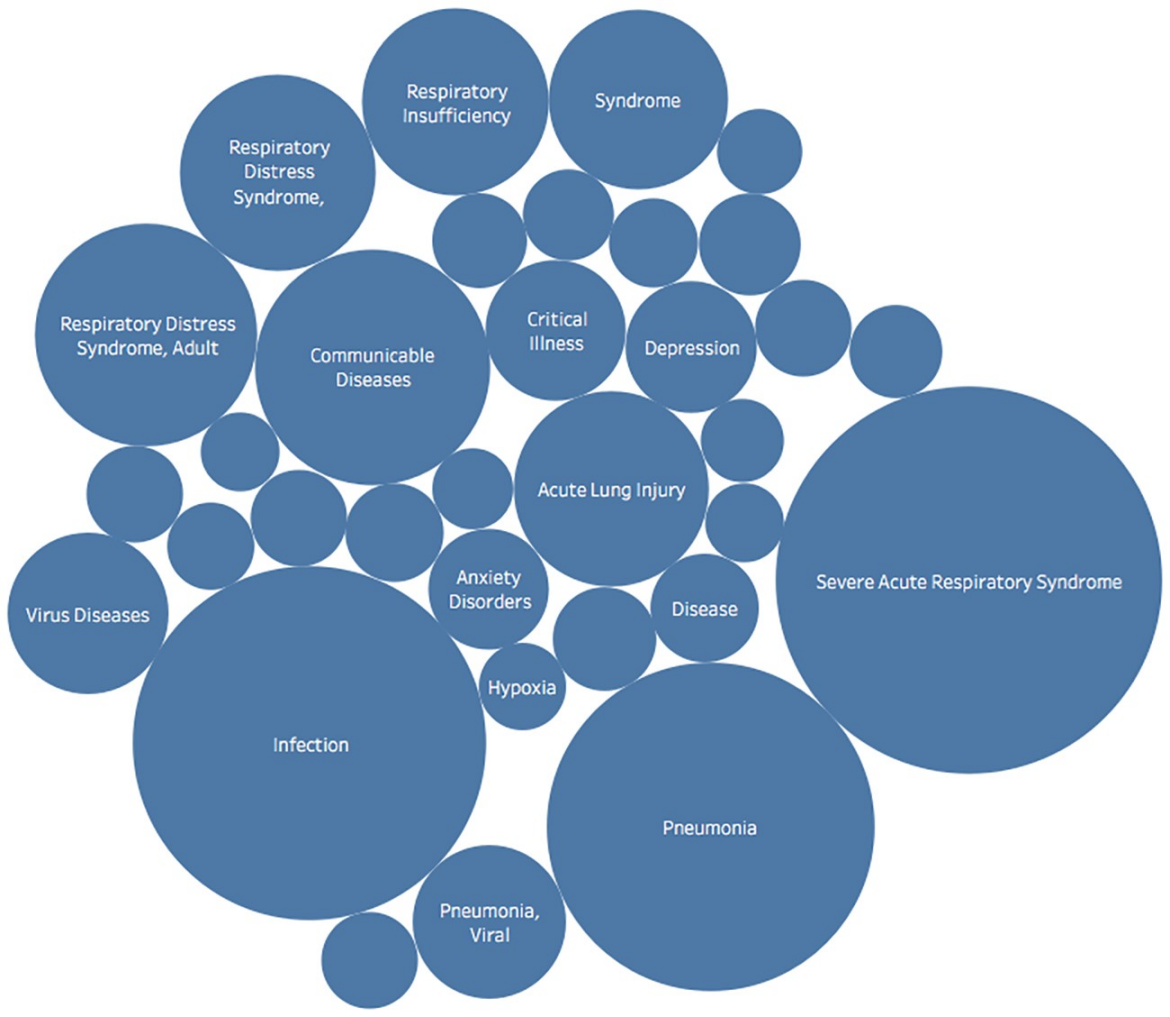
MeSH information details the most prevalent MeSH terms across COVID-19 related clinical trials. Some notable and expected prominent terms are Infections, Severe Acute Respiratory Syndrome, and Pneumonia.

Across COVID-19 related clinical trials, the most prevalent MeSH terms, as illustrated in Fig 7, are Infections, Severe Acute Respiratory Syndrome, and Pneumonia. The frequencies of MeSH terms shed light on the areas researchers are currently examining and possibly illuminate under-researched topics.

**Fig 8:**
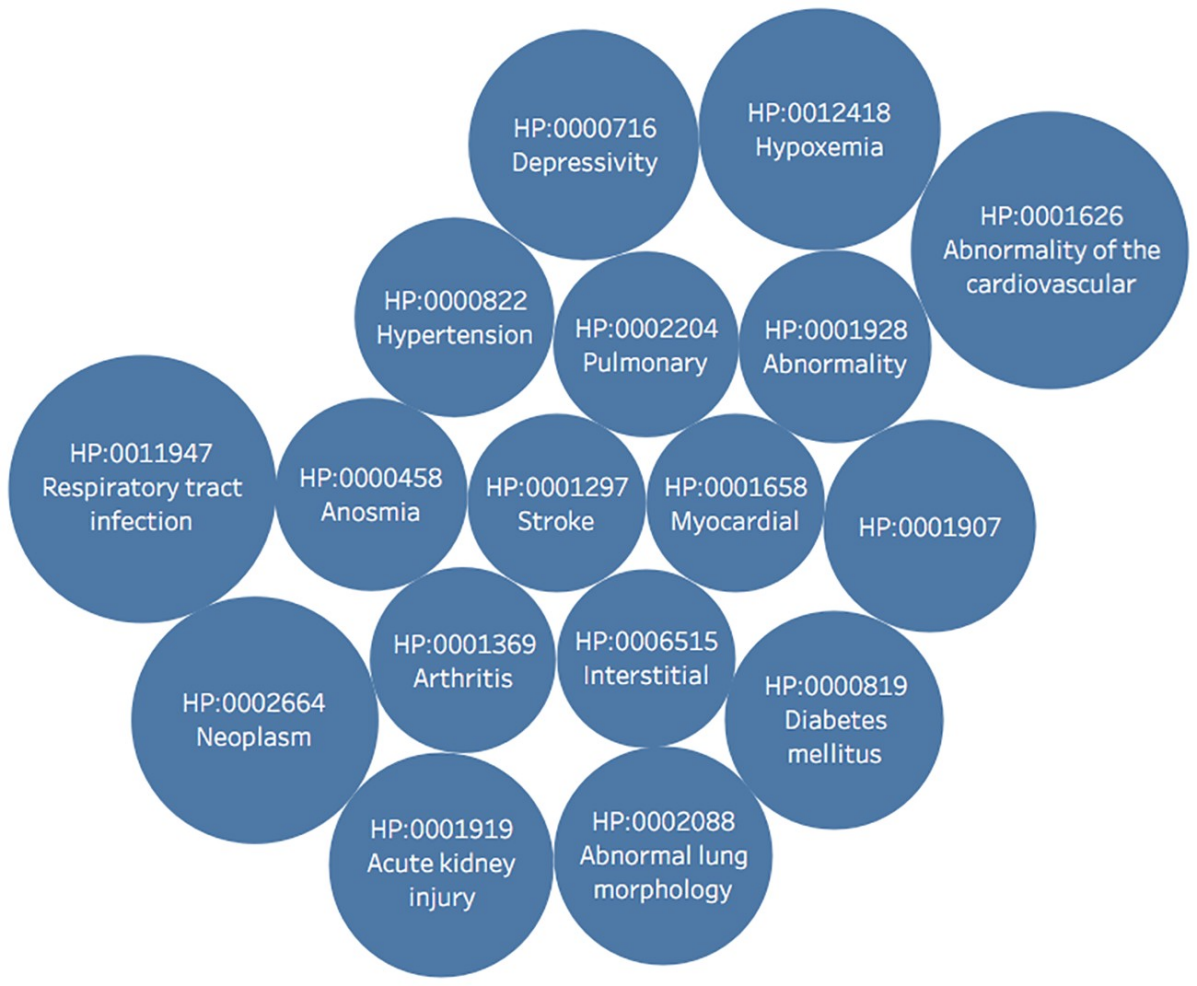
HPO information portrays the most widely noted HPO terms. Note that HPO terms are normalized as detailed in Alag 2020 [6]. The most frequent terms were Respiratory tract infection, Abnormality of the cardiovascular system, Acute kidney injury, Abnormal lung morphology, Depressivity, Diabetes mellitus, and more.

As illustrated in Fig 8, Respiratory tract infection and Abnormality of the cardiovascular system are the leading HPO nodes that were identified with COVID-19 trials. The validity of our methodology is upheld by the occurrence of HPO nodes like Abnormal lung morphology, Abnormality of the cardiovascular system, Acute kidney injury, and Respiratory HPO terms since these terms have proven to be associated with COVID-19.

#### Reports for drugs, MeSH, and HPO terms associated with COVID-19 clinical trials

An HTML report was created for each of the unique drugs, MeSH, and HPO terms associated with COVID-19 clinical trials. Longitudinal access (reports over multiple time periods) is freely available via the home page (http://CovidResearchTrials.com). As shown in S1 File, each report contains a list of either the drug, the MeSH terms, or the HPO terms. All of the terms in a category are displayed on the left-hand side of the report to enable easy navigation, and the reports contain a list of correlated drugs, MeSH, and HPO terms. Further, all reports contain the details of the clinical trials in which the term is referenced. Every clinical trial report shows the mapped HPO and MeSH terms, which are also hyperlinked. Related HPO terms, with their associated genes, protein mutations, and SNPs are also referenced in the report.

### Correlations across drugs, MeSH, and HPO terms

In this section, two representative terms are selected to demonstrate the results of the clustering across drugs, MeSH, and HPO terms. Terms related to the MeSH term “D018352: Coronavirus Infections” are shown in Table 1 and discussed below:

1. **Drugs**: As expected, the most common drugs include comparisons to Placebo, Hydroxychloroquine [17], Standard of care, No Intervention, Ivermectin [18], Nitazoxanide [19], Favipiravir [20], Remdesivir [21], Vitamin C, etc. Since these drugs are all related to COVID-19 efforts, the methodology for correlation is validated, suggesting the potential of using more advanced machine learning techniques.
2. **MeSH**: Related MeSH terms are D045169 Severe Acute Respiratory Syndrome, D003141 Communicable Disease, D007239 Infection, D011014 Pneumonia, D013577 Syndrome, D012128 Respiratory Distress Syndrome, Adult, D012127 Respiratory Distress Syndrome, Newborn, D014777 Virus Diseases, D055371 Acute Lung Injury, D011024 Pneumonia, Viral, D003333 Coronaviridae Infections, D012141 Respiratory Tract Infections, D012327 RNA Virus Infections, D016638 Critical Illness, etc. As COVID-19 is a communicable disease, caused by a virus that causes an infection associated with the respiratory system, the appearance of as a top MeSH term only increases the validity of the methdology [22].
3. **HPO Terms**: Related HPO terms include: HP:0002090 Pneumonia, HP:0011947 Respiratory tract infection, HP:0000819 Diabetes mellitus, HP:0010444 Pulmonary insufficiency, HP:0012418 Hypoxemia, HP:0002905 Hyperphosphatemia, HP:0006802 Abnormal anterior horn cell morphology, HP:0001871 Abnormality of blood and blood-forming tissues, HP:0002900 Hypokalemia, HP:0000846 Adrenal insufficiency, HP:0000132 Menorrhagia, HP:0002088 Abnormal lung morphology, HP:0005978 Type II diabetes mellitus, etc. The correlation algorithm can even discern diabetes as related to COVID-19, which is accurate as individuals with diabetes are at an increased risk of developing severe illness from COVID-19 [23].

**Table 1:** Related drugs, MeSH, and HPO terms using co-occurrences with D018352: Coronavirus infection.

	Term Type	Related Term	Score
1	Drug	Placebo [multiple]	0.62
		Hydroxychloroquine	0.19
		No intervention	0.12
		Ivermectin	0.11
		Nitazoxanide	0.10
		Favipiravir	0.10
		Remdesivir	0.10
		Vitamin C	0.09
		Zinc	0.09
		Rivaroxaban	0.09
2	MeSH	D045169 Severe Acute Respiratory Syndrome	0.8
		D003141 Communicable Disease	0.28
		D007239 Infection	0.27
		D011014 Pneumonia	0.18
		D013577 Syndrome	0.17
		D012128 Respiratory Distress Syndrome, Adult	0.13
		D012127 Respiratory Distress Syndrome, Newborn	0.13
		D014777 Virus Diseases	0.13
		D055371 Acute Lung Injury	0.12
		D011024 Pneumonia, Viral	0.11
		D003333 Coronaviridae Infections	0.09
		D012141 Respiratory Tract Infections	0.07
		D012327 RNA Virus Infections	0.07
		D016638 Critical Illness	0.06
3	HPO	HP:0002090 Pneumonia	0.18
		HP:0011947 Respiratory tract infection	0.08
		HP:0000819 Diabetes mellitus	0.05
		HP:0010444 Pulmonary insufficiency	0.04
		HP:0012418 Hypoxemia	0.04
		HP:0002905 Hyperphosphatemia	0.04
		HP:0006802 Abnormal anterior horn cell morphology	0.04
		HP:0001871 Abnormality of blood and blood-forming tissues	0.04
		HP:0002900 Hypokalemia	0.04
		HP:0000846 Adrenal insufficiency	0.04
		HP:0000132 Menorrhagia	0.04
		HP:0002088 Abnormal lung morphology	0.04
		HP:0005978 Type II diabetes mellitus	0.04

Results from finding similar Drugs, MeSH, and HPO terms for the MeSH term Coronavirus Infection (from August 2020). For full details and updated results readers are referred to the report that is available at http://CovidResearchTrials.com.

Similarly, terms related to the drug Hydroxychloroquine [17] are shown in Table 2 and discussed below:

1. **Drugs**: Other drugs correlated to Hydroxychloroquine are: Azithromycin, Placebo, Interferon Beta-1A, Routine care for COVID-19 patients, Lopinavir-Ritonavir, Favipiravir, Interferon Beta-1B, Zinc, Vitamin D, etc.
2. **MeSH**: Related MeSH terms are D003141 Communicable Diseases, D007239 Infection, D045169 Severe Acute Respiratory Syndrome, D055371 Acute Lung Injury, D018352 Coronavirus Infections, D012127 Respiratory Distress Syndrome, Newborn, D012128 Respiratory Distress Syndrome, Adult, etc.
3. **HPO Terms**: Related HPO terms include: HP:0003326 Myalgia, HP:0002090 Pneumonia, HP:0100526 Neoplasm of the lung, HP:0002487 Hyperkinetic movements, HP:0100598 Pulmonary edema, HP:0030358 Non-small cell lung carcinoma, etc.

**Table 2:** Related drugs, MeSH, and HPO terms using co-occurrences with drug: Hydroxychloroquine.

	Term Type	Related Term	Score
1	Drug	Azithromycin	0.37
		Placebo [multiple]	0.22
		Vitamin C	0.17
		Interferon Beta-1A	0.15
		Therapeutic anticoagulation	0.14
		Routine care for COVID-19 patients	0.14
		Lopinavir-Ritonavir	0.14
		Favipiravir	0.17
		Interferon Beta-1B	0.11
		Zinc	0.11
		Vitamin D	0.11
2	MeSH	D003141 Communicable Diseases	0.27
		D007239 Infection	0.26
		D045169 Severe Acute Respiratory Syndrome	0.21
		D055371 Acute Lung Injury	0.19
		D018352 Coronavirus Infections	0.19
		D012127 Respiratory Distress Syndrome, Newborn	0.17
		D012128 Respiratory Distress Syndrome, Adult	0.16
3	HPO	HP:0003326 Myalgia	0.10
		HP:0002090 Pneumonia	0.09
		HP:0100526 Neoplasm of the lung	0.09
		HP:0002487 Hyperkinetic movements	0.07
		HP:0100598 Pulmonary edema	0.07
		HP:0030358 Non-small cell lung carcinoma	0.07

Results from finding similar Drugs, MeSH, and HPO terms for the Drug: Hydroxychloroquine (August 2020). For full details and latest results readers are referred to the report that is available at http://CovidResearchTrials.com

Readers are encouraged to see the full results and many other correlations/insights from the project home page http://CovidResearchTrials.com.

## Conclusion and future work

In this work, COVID-19 related clinical trials were not only successfully mined from ClinicalTrials.gov but also associated with Drugs, HPO, and MeSH terms. Unique reports for intervention/drugs, MeSH, and HPO terms were created and are freely available on the web, along with APIs (Java and Google Colab notebooks) for programmatic access. Further, the publicly-available site (http://CovidResearchTrials.com) contains analysis at multiple time points, further providing researchers with longitudinal information about clinical trials and associated entities, as well as demonstrating the reproducibility of the methods. The programmatic access of the data connecting COVID-19 with MeSH and HPO terms can also be useful for machine learning and other insights. This methodology and the generated reports provide a succinct summary of COVID-19 related Interventions/Drugs, MeSH terms, HPO terms, clinical trials, genes, SNPs, and protein mutations all in one place. Overall, the insights and resources generated could potentially be an invaluable, time-saving resource to researchers, clinicians, and the lay public.

In the future, this framework can additionally be applied to other scientific corpora, such as PubMed [24] and PubMed Central [25]. Further, as evaluations will be done at future timepoints, the changes in trends over the coming months will be noteworthy and may provide insight onto the global community united response to fight the COVID-19 pandemic. The analysis results can be furthered enhanced by normalizing drug/intervention terms across the clinical trials.

## Supporting information

The following supporting figures are available for this article:

S1 File Screen shots of Covid Research Trials homepage, various reports, and API tool kits. (PDF)

S2 File Screen shots of Covid Research Trials Java API documentation. (PDF)
