# Supplementary material for: Analysis of COVID-19 clinical trials: A data-driven, ontology-based, and natural language processing approach
Source: PLoS One. 2020 Sep 30;15(9):e0239694. doi: 10.1371/journal.pone.0239694 (PMC7526926; doi:10.1371/journal.pone.0239694)
Supplement: S1 File — (PDF) [file pone.0239694.s001.pdf]

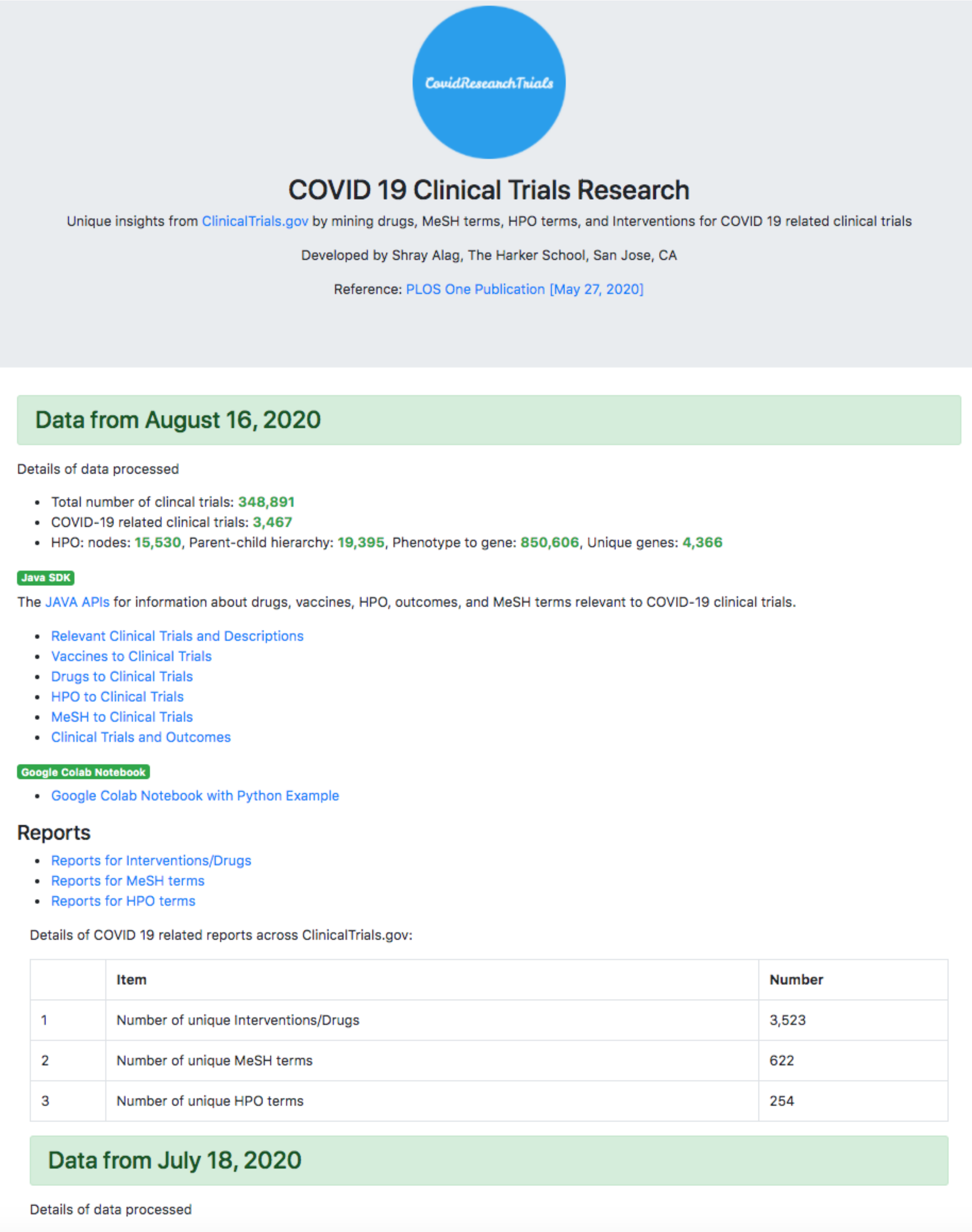

CovidResearchTrials

COVID 19 Clinical Trials Research

Unique insights from ClinicalTrials.gov by mining drugs, MeSH terms, HPO terms, and Interventions for COVID 19 related clinical trials

Developed by Shray Alag, The Harker School, San Jose, CA

Reference: PLOS One Publication [May 27, 2020]

Data from August 16, 2020

Details of data processed

- Total number of clincal trials: **348,891**
- COVID-19 related clinical trials: **3,467**
- HPO: nodes: **15,530**, Parent-child hierarchy: **19,395**, Phenotype to gene: **850,606**, Unique genes: **4,366**

Java SDK

The JAVA APIs for information about drugs, vaccines, HPO, outcomes, and MeSH terms relevant to COVID-19 clinical trials.

- Relevant Clinical Trials and Descriptions
- Vaccines to Clinical Trials
- Drugs to Clinical Trials
- HPO to Clinical Trials
- MeSH to Clinical Trials
- Clinical Trials and Outcomes

Google Colab Notebook

- Google Colab Notebook with Python Example

Reports

- Reports for Interventions/Drugs
- Reports for MeSH terms
- Reports for HPO terms

Details of COVID 19 related reports across ClinicalTrials.gov:

	Item	Number
1	Number of unique Interventions/Drugs	3,523
2	Number of unique MeSH terms	622
3	Number of unique HPO terms	254

Data from July 18, 2020

Details of data processed

Figure 1. Screenshot of the home page of Covid-19 Research Trials (http://covidresearchtrials.com), which provides access to analytics, reports, and APIs.

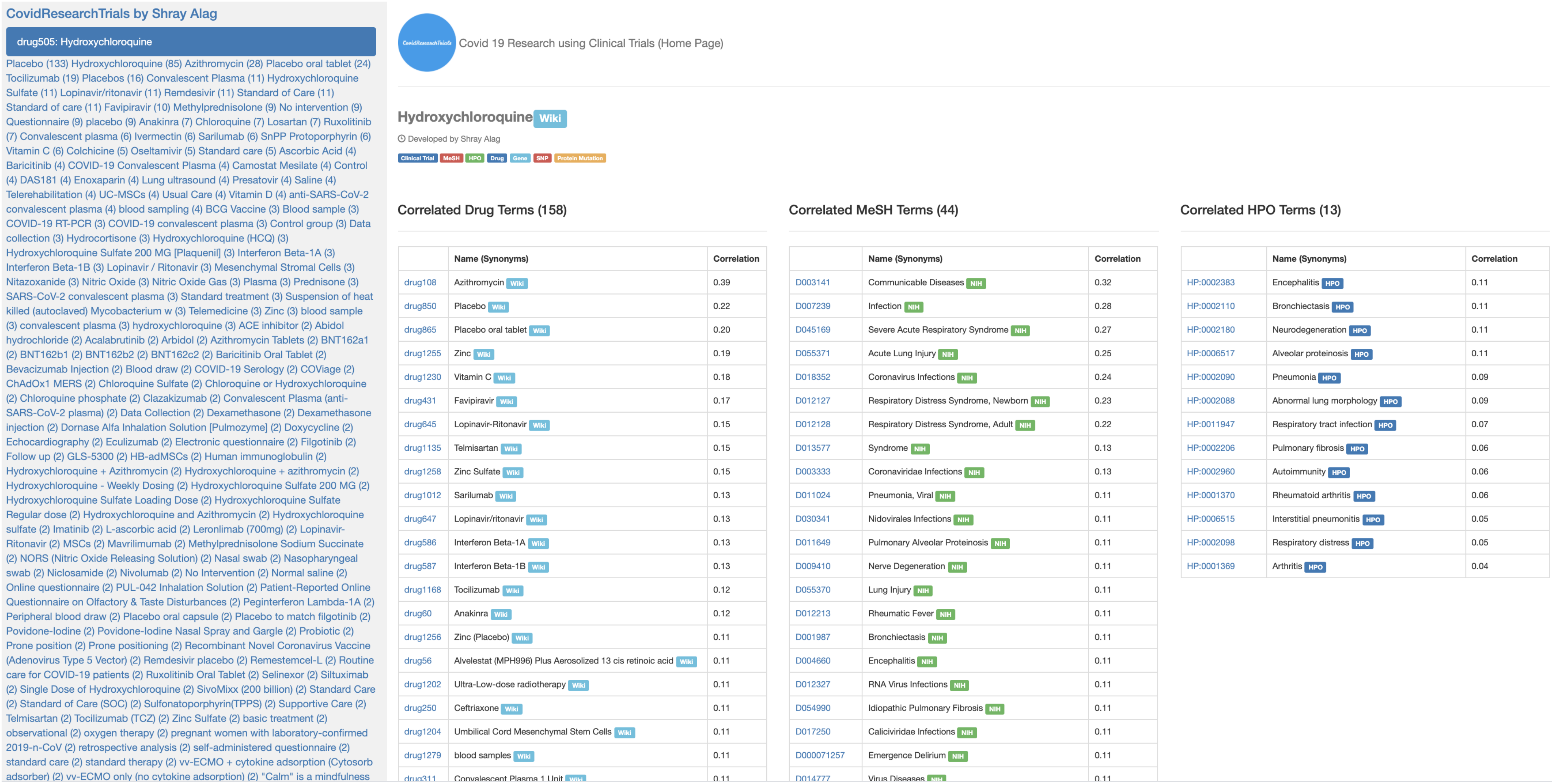

Figure 2. Screenshot of the top part of the report related to a Drug with related MeSH and HPO nodes.

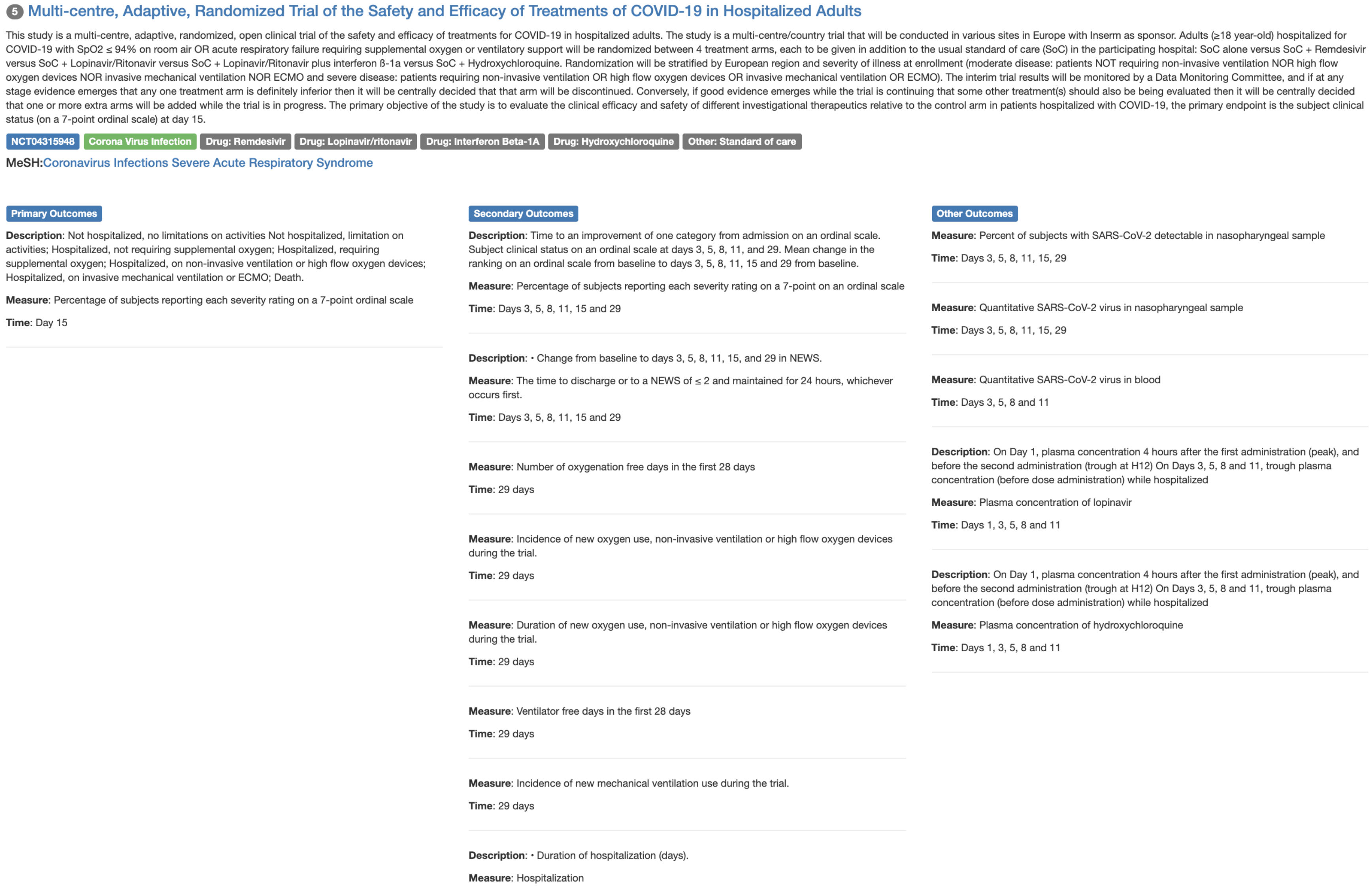

⑤ Multi-centre, Adaptive, Randomized Trial of the Safety and Efficacy of Treatments of COVID-19 in Hospitalized Adults

This study is a multi-centre, adaptive, randomized, open clinical trial of the safety and efficacy of treatments for COVID-19 in hospitalized adults. The study is a multi-centre/country trial that will be conducted in various sites in Europe with Inserm as sponsor. Adults (≥18 year-old) hospitalized for COVID-19 with SpO2 ≤ 94% on room air OR acute respiratory failure requiring supplemental oxygen or ventilatory support will be randomized between 4 treatment arms, each to be given in addition to the usual standard of care (SoC) in the participating hospital: SoC alone versus SoC + Remdesivir versus SoC + Lopinavir/Ritonavir versus SoC + Lopinavir/Ritonavir plus interferon ß-1a versus SoC + Hydroxychloroquine. Randomization will be stratified by European region and severity of illness at enrollment (moderate disease: patients NOT requiring non-invasive ventilation NOR high flow oxygen devices NOR invasive mechanical ventilation NOR ECMO and severe disease: patients requiring non-invasive ventilation OR high flow oxygen devices OR invasive mechanical ventilation OR ECMO). The interim trial results will be monitored by a Data Monitoring Committee, and if at any stage evidence emerges that any one treatment arm is definitely inferior then it will be centrally decided that that arm will be discontinued. Conversely, if good evidence emerges while the trial is continuing that some other treatment(s) should also be being evaluated then it will be centrally decided that one or more extra arms will be added while the trial is in progress. The primary objective of the study is to evaluate the clinical efficacy and safety of different investigational therapeutics relative to the control arm in patients hospitalized with COVID-19, the primary endpoint is the subject clinical status (on a 7-point ordinal scale) at day 15.

NCT04315948 | Corona Virus Infection | Drug: Remdesivir | Drug: Lopinavir/ritonavir | Drug: Interferon Beta-1A | Drug: Hydroxychloroquine | Other: Standard of care

MeSH: Coronavirus Infections Severe Acute Respiratory Syndrome

Primary Outcomes

Description: Not hospitalized, no limitations on activities Not hospitalized, limitation on activities; Hospitalized, not requiring supplemental oxygen; Hospitalized, requiring supplemental oxygen; Hospitalized, on non-invasive ventilation or high flow oxygen devices; Hospitalized, on invasive mechanical ventilation or ECMO; Death.

Measure: Percentage of subjects reporting each severity rating on a 7-point ordinal scale

Time: Day 15

Secondary Outcomes

Description: Time to an improvement of one category from admission on an ordinal scale. Subject clinical status on an ordinal scale at days 3, 5, 8, 11, and 29. Mean change in the ranking on an ordinal scale from baseline to days 3, 5, 8, 11, 15 and 29 from baseline.

Measure: Percentage of subjects reporting each severity rating on a 7-point on an ordinal scale

Time: Days 3, 5, 8, 11, 15 and 29

Description: • Change from baseline to days 3, 5, 8, 11, 15, and 29 in NEWS.

Measure: The time to discharge or to a NEWS of ≤ 2 and maintained for 24 hours, whichever occurs first.

Time: Days 3, 5, 8, 11, 15 and 29

Measure: Number of oxygenation free days in the first 28 days

Time: 29 days

Measure: Incidence of new oxygen use, non-invasive ventilation or high flow oxygen devices during the trial.

Time: 29 days

Measure: Duration of new oxygen use, non-invasive ventilation or high flow oxygen devices during the trial.

Time: 29 days

Measure: Ventilator free days in the first 28 days

Time: 29 days

Measure: Incidence of new mechanical ventilation use during the trial.

Time: 29 days

Description: • Duration of hospitalization (days).

Measure: Hospitalization

Other Outcomes

Measure: Percent of subjects with SARS-CoV-2 detectable in nasopharyngeal sample

Time: Days 3, 5, 8, 11, 15, 29

Measure: Quantitative SARS-CoV-2 virus in nasopharyngeal sample

Time: Days 3, 5, 8, 11, 15, 29

Measure: Quantitative SARS-CoV-2 virus in blood

Time: Days 3, 5, 8 and 11

Description: On Day 1, plasma concentration 4 hours after the first administration (peak), and before the second administration (trough at H12) On Days 3, 5, 8 and 11, trough plasma concentration (before dose administration) while hospitalized

Measure: Plasma concentration of lopinavir

Time: Days 1, 3, 5, 8 and 11

Description: On Day 1, plasma concentration 4 hours after the first administration (peak), and before the second administration (trough at H12) On Days 3, 5, 8 and 11, trough plasma concentration (before dose administration) while hospitalized

Measure: Plasma concentration of hydroxychloroquine

Time: Days 1, 3, 5, 8 and 11

Figure 3. Screenshot of a Clinical Trial referenced in the report.

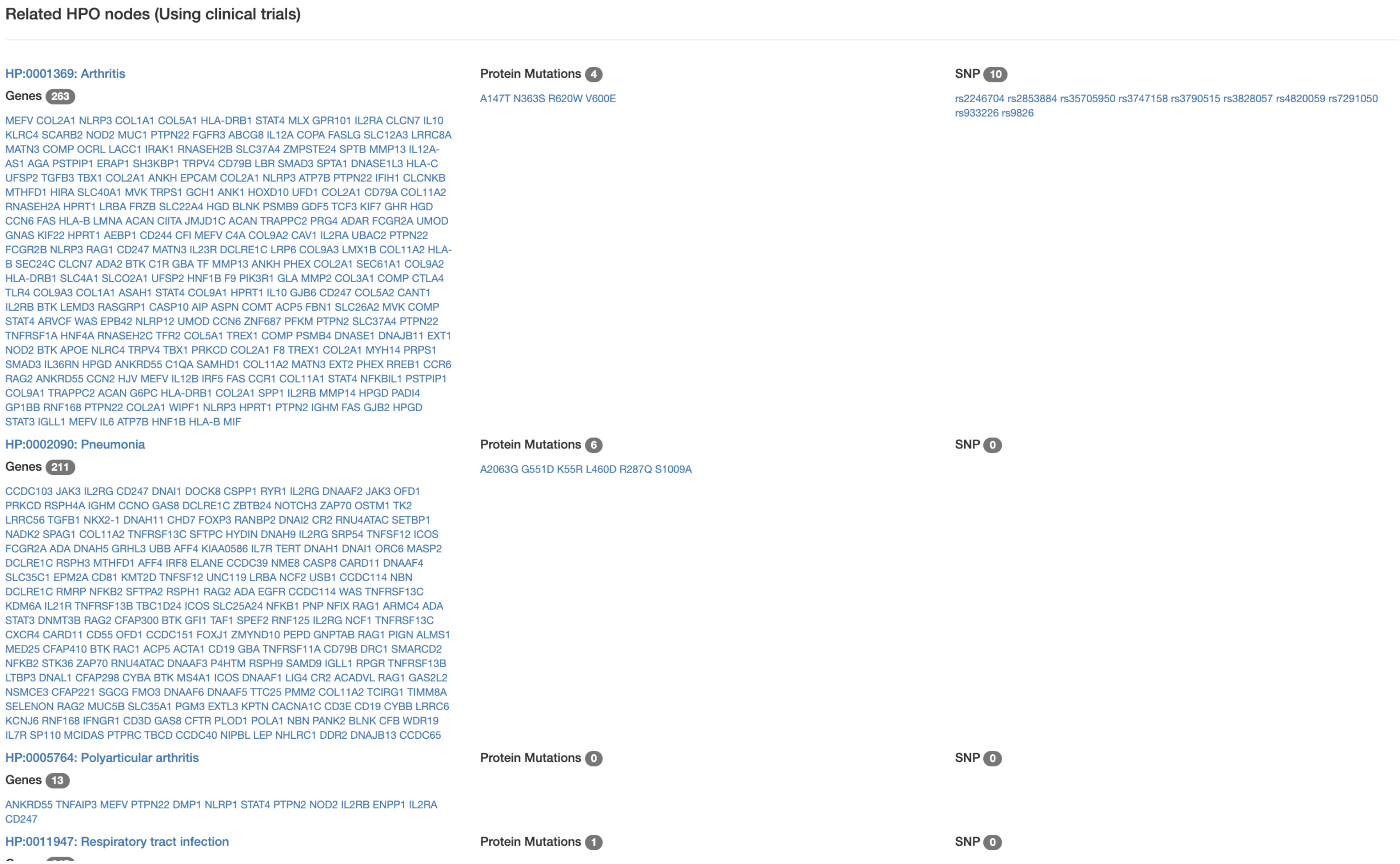

Related HPO nodes (Using clinical trials)

HP:0001369: Arthritis

Genes 263

MEFV COL2A1 NLRP3 COL1A1 COL5A1 HLA-DRB1 STAT4 MLX GPR101 IL2RA CLCN7 IL10 KLRC4 SCARB2 NOD2 MUC1 PTPN22 FGFR3 ABCG8 IL12A COPA FASLG SLC12A3 LRRC8A MATN3 COMP OCRL LACC1 IRAK1 RNASEH2B SLC37A4 ZMPSTE24 SPTB MMP13 IL12A-AS1 AGA PSTPIP1 ERAP1 SH3KBP1 TRPV4 CD79B LBR SMAD3 SPTA1 DNASE1L3 HLA-C UFSP2 TGFB3 TBX1 COL2A1 ANKH EPCAM COL2A1 NLRP3 ATP7B PTPN22 IFIH1 CLCNKB MTHFD1 HIRA SLC40A1 MVK TRPS1 GCH1 ANK1 HOXD10 UFD1 COL2A1 CD79A COL11A2 RNASEH2A HPRT1 LRBA FRZB SLC22A4 HGD BLNK PSMB9 GDF5 TCF3 KIF7 GHR HGD CCN6 FAS HLA-B LMNA ACAN CIITA JMJD1C ACAN TRAPPC2 PRG4 ADAR FCGR2A UMOD GNAS KIF22 HPRT1 AEBP1 CD244 CFI MEFV C4A COL9A2 CAV1 IL2RA UBAC2 PTPN22 FCGR2B NLRP3 RAG1 CD247 MATN3 IL23R DCLRE1C LRP6 COL9A3 LMX1B COL11A2 HLA-B SEC24C CLCN7 ADA2 BTK C1R GBA TF MMP13 ANKH PHEX COL2A1 SEC61A1 COL9A2 HLA-DRB1 SLC4A1 SLCO2A1 UFSP2 HNF1B F9 PIK3R1 GLA MMP2 COL3A1 COMP CTLA4 TLR4 COL9A3 COL1A1 ASAH1 STAT4 COL9A1 HPRT1 IL10 GJB6 CD247 COL5A2 CANT1 IL2RB BTK LEMD3 RASGRP1 CASP10 AIP ASPN COMT ACP5 FBN1 SLC26A2 MVK COMP STAT4 ARVCF WAS EPB42 NLRP12 UMOD CCN6 ZNF687 PFKM PTPN2 SLC37A4 PTPN22 TNFRSF1A HNF4A RNASEH2C TFR2 COL5A1 TREX1 COMP PSMB4 DNASE1 DNAJB11 EXT1 NOD2 BTK APOE NLRC4 TRPV4 TBX1 PRKCD COL2A1 F8 TREX1 COL2A1 MYH14 PRPS1 SMAD3 IL36RN HPGD ANKRD55 C1QA SAMHD1 COL11A2 MATN3 EXT2 PHEX RREB1 CCR6 RAG2 ANKRD55 CCN2 HJV MEFV IL12B IRF5 FAS CCR1 COL11A1 STAT4 NFKBIL1 PSTPIP1 COL9A1 TRAPPC2 ACAN G6PC HLA-DRB1 COL2A1 SPP1 IL2RB MMP14 HPGD PADI4 GP1BB RNF168 PTPN22 COL2A1 WIPF1 NLRP3 HPRT1 PTPN2 IGHM FAS GJB2 HPGD STAT3 IGLL1 MEFV IL6 ATP7B HNF1B HLA-B MIF

Protein Mutations 4

A147T N363S R620W V600E

SNP 10

rs2246704 rs2853884 rs35705950 rs3747158 rs3790515 rs3828057 rs4820059 rs7291050 rs933226 rs9826

HP:0002090: Pneumonia

Genes 211

CCDC103 JAK3 IL2RG CD247 DNAI1 DOCK8 CSPP1 RYR1 IL2RG DNAAF2 JAK3 OFD1 PRKCD RSPH4A IGHM CCNO GAS8 DCLRE1C ZBTB24 NOTCH3 ZAP70 OSTM1 TK2 LRRC56 TGFB1 NKX2-1 DNAH11 CHD7 FOXP3 RANBP2 DNAI2 CR2 RNU4ATAC SETBP1 NADK2 SPAG1 COL11A2 TNFRSF13C SFTPC HYDIN DNAH9 IL2RG SRP54 TNFSF12 ICOS FCGR2A ADA DNAH5 GRHL3 UBB AFF4 KIAA0586 IL7R TERT DNAH1 DNAI1 ORC6 MASP2 DCLRE1C RSPH3 MTHFD1 AFF4 IRF8 ELANE CCDC39 NME8 CASP8 CARD11 DNAAF4 SLC35C1 EPM2A CD81 KMT2D TNFSF12 UNC119 LRBA NCF2 USB1 CCDC114 NBN DCLRE1C RMRP NFKB2 SFTPA2 RSPH1 RAG2 ADA EGFR CCDC114 WAS TNFRSF13C KDM6A IL21R TNFRSF13B TBC1D24 ICOS SLC25A24 NFKB1 PNP NFIX RAG1 ARMC4 ADA STAT3 DNMT3B RAG2 CFAP300 BTK GFI1 TAF1 SPEF2 RNF125 IL2RG NCF1 TNFRSF13C CXCR4 CARD11 CD55 OFD1 CCDC151 FOXJ1 ZMYND10 PEPD GNPTAB RAG1 PIGN ALMS1 MED25 CFAP410 BTK RAC1 ACP5 ACTA1 CD19 GBA TNFRSF11A CD79B DRC1 SMARCD2 NFKB2 STK36 ZAP70 RNU4ATAC DNAAF3 P4HTM RSPH9 SAMD9 IGLL1 RPGR TNFRSF13B LTBP3 DNAL1 CFAP298 CYBA BTK MS4A1 ICOS DNAAF1 LIG4 CR2 ACADVL RAG1 GAS2L2 NSMCE3 CFAP221 SGCG FMO3 DNAAF6 DNAAF5 TTC25 PMM2 COL11A2 TCIRG1 TIMM8A SELENON RAG2 MUC5B SLC35A1 PGM3 EXTL3 KPTN CACNA1C CD3E CD19 CYBB LRRC6 KCNJ6 RNF168 IFNGR1 CD3D GAS8 CFTR PLOD1 POLA1 NBN PANK2 BLNK CFB WDR19 IL7R SP110 MCIDAS PTPRC TBCD CCDC40 NIPBL LEP NHLRC1 DDR2 DNAJB13 CCDC65

Protein Mutations 6

A2063G G551D K55R L460D R287Q S1009A

SNP 0

HP:0005764: Polyarticular arthritis

Genes 13

ANKRD55 TNFAIP3 MEFV PTPN22 DMP1 NLRP1 STAT4 PTPN2 NOD2 IL2RB ENPP1 IL2RA CD247

Protein Mutations 0

SNP 0

HP:0011947: Respiratory tract infection

Protein Mutations 1

SNP 0

Figure 4. Screenshot of the HPO section at the bottom of the report. For each HPO node it has the associated Genes, SNPs, and Protein Mutations.

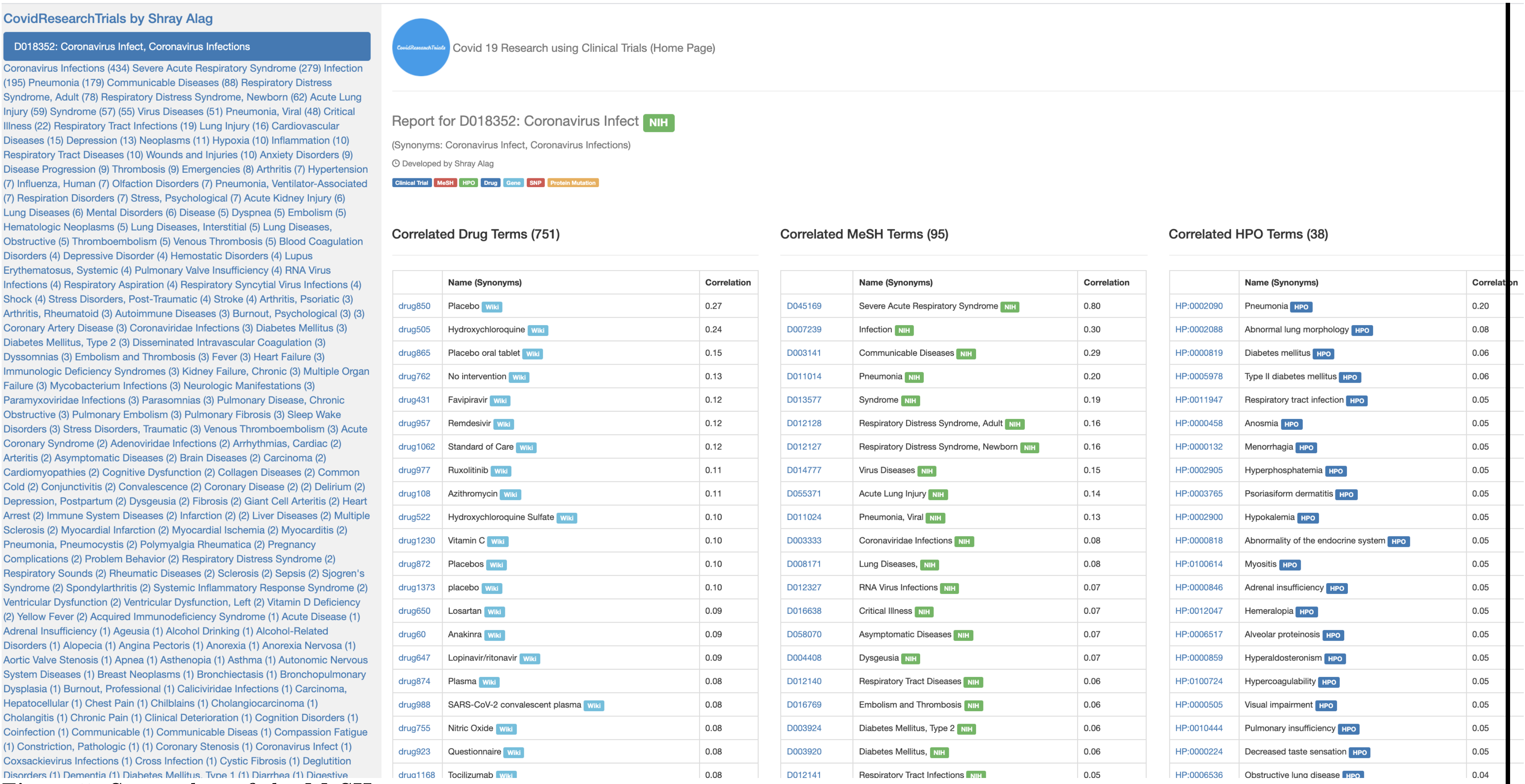

Figure 5. Screenshot of the MeSH report.

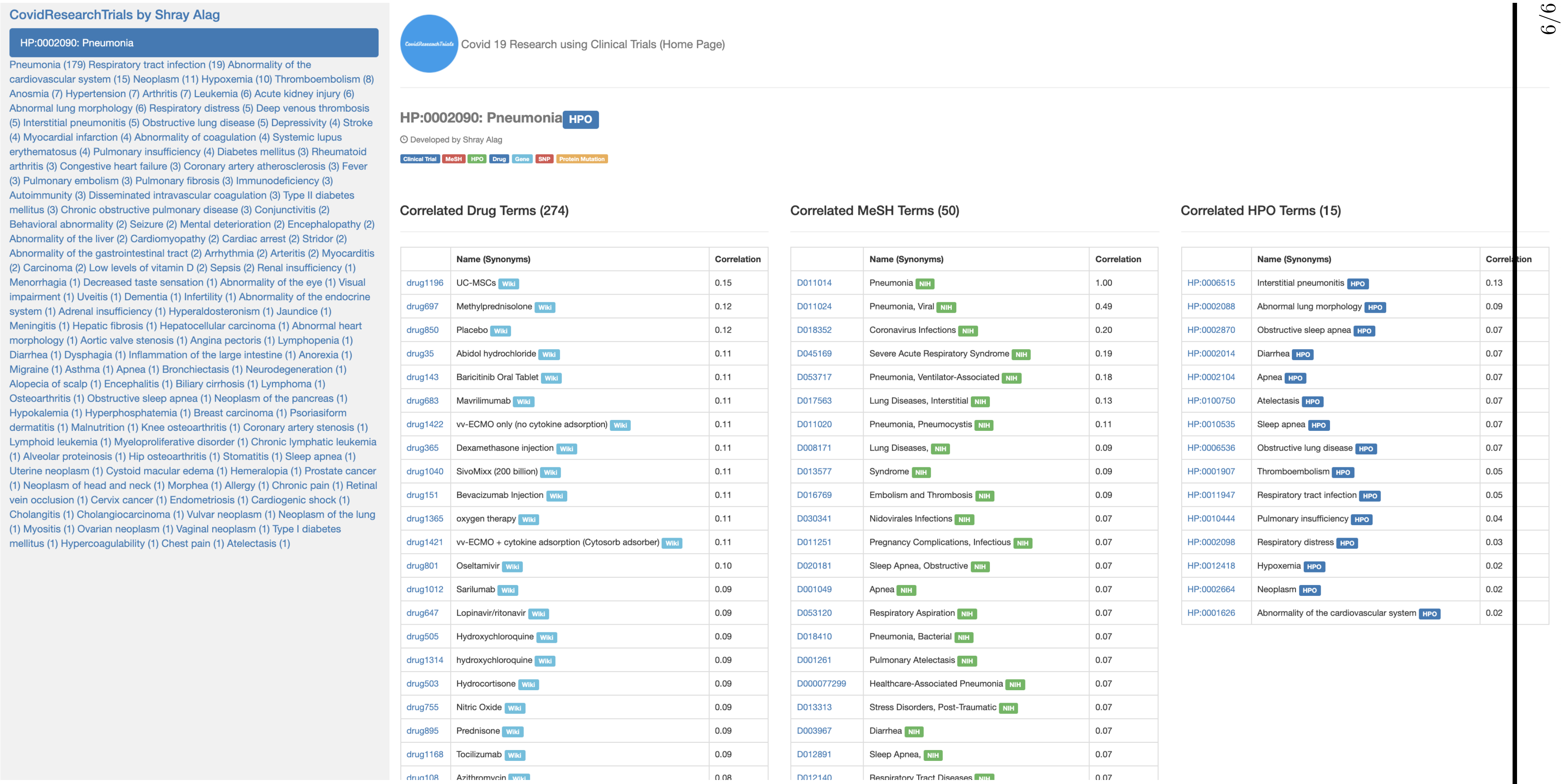

Figure 6. Screenshot of the HPO report.
