# Supplementary material for: Analysis of COVID-19 clinical trials: A data-driven, ontology-based, and natural language processing approach
Source: PLoS One. 2020 Sep 30;15(9):e0239694. doi: 10.1371/journal.pone.0239694 (PMC7526926; doi:10.1371/journal.pone.0239694)
Supplement: S2 File — (PDF) [file pone.0239694.s002.pdf]

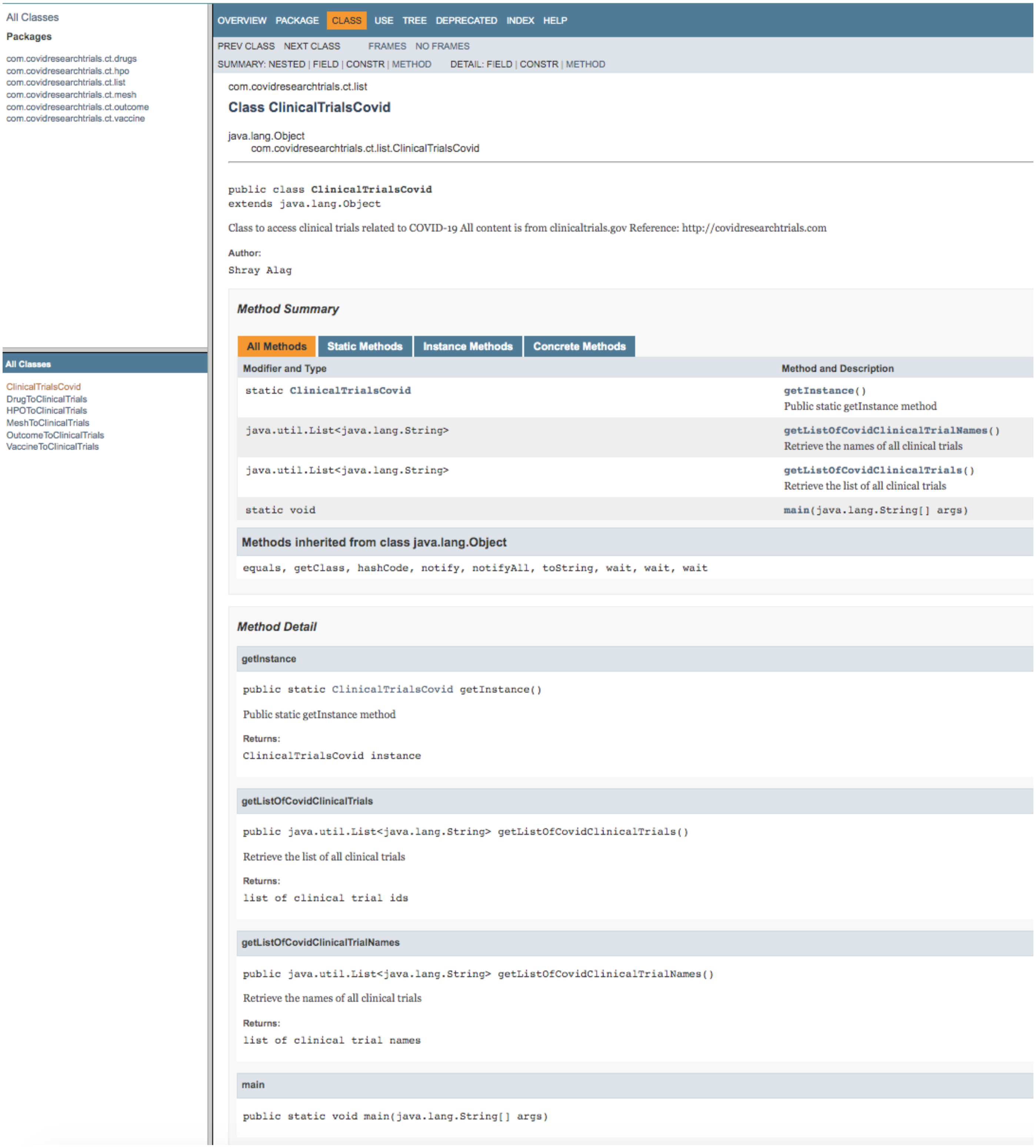

Figure 1. Screenshot of the API page for accessing the list of COVID-19 clinical trials (http://covidresearchtrials.com/08162020/api/doc/index.html). Class: com.covidresearchtrials.ct.list.ClinicalTrialsCovid

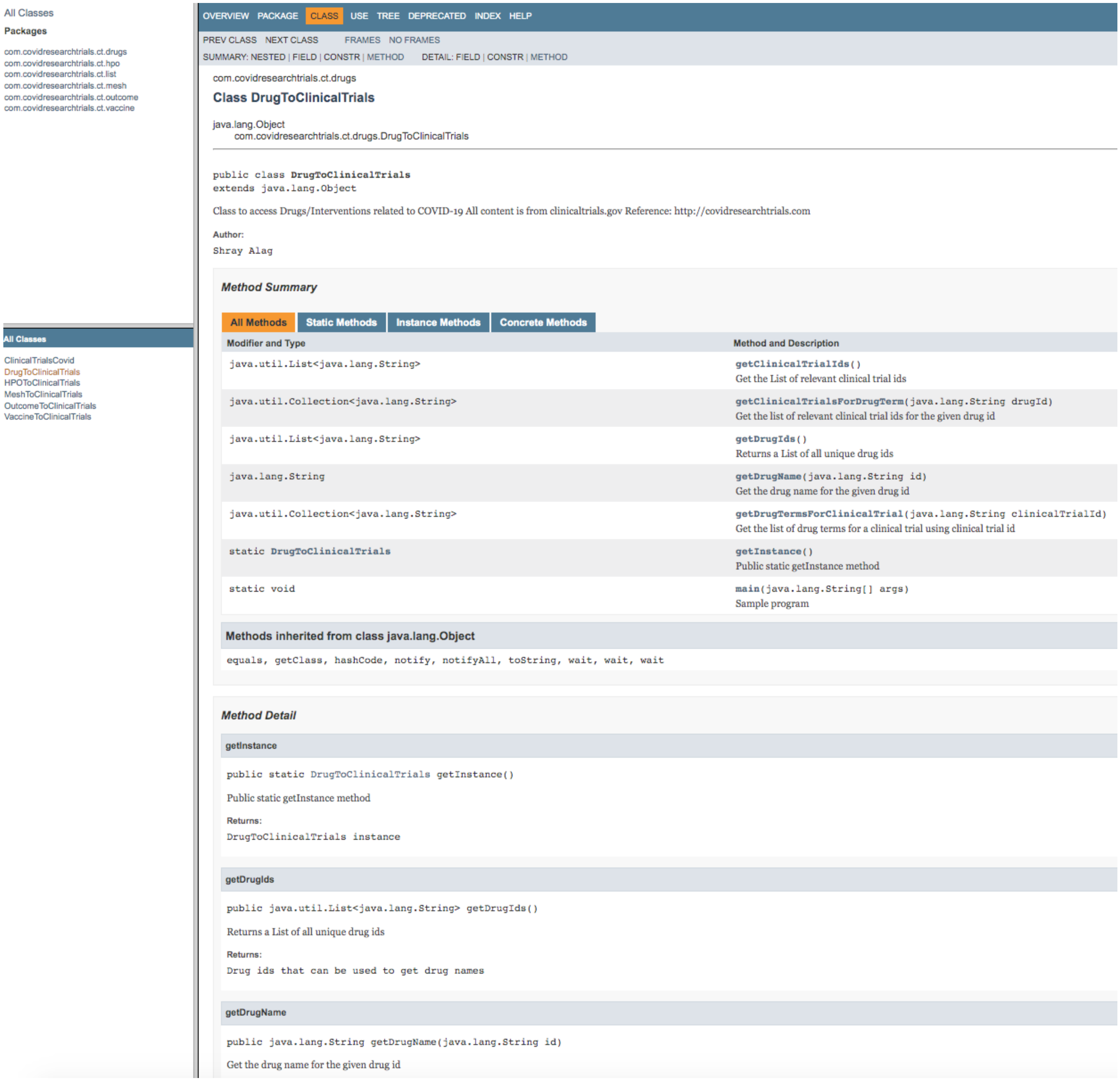

All Classes

Packages

com.covidresearchtrials.ct.drugs
com.covidresearchtrials.ct.hpo
com.covidresearchtrials.ct.list
com.covidresearchtrials.ct.mesh
com.covidresearchtrials.ct.outcome
com.covidresearchtrials.ct.vaccine

All Classes

ClinicalTrialsCovid
DrugToClinicalTrials
HPOToClinicalTrials
MeshToClinicalTrials
OutcomeToClinicalTrials
VaccineToClinicalTrials

OVERVIEW PACKAGE CLASS USE TREE DEPRECATED INDEX HELP

PREV CLASS NEXT CLASS FRAMES NO FRAMES
SUMMARY: NESTED | FIELD | CONSTR | METHOD DETAIL: FIELD | CONSTR | METHOD

com.covidresearchtrials.ct.drugs

Class DrugToClinicalTrials

java.lang.Object
com.covidresearchtrials.ct.drugs.DrugToClinicalTrials

```
public class DrugToClinicalTrials
extends java.lang.Object
```

Class to access Drugs/Interventions related to COVID-19 All content is from clinicaltrials.gov Reference: http://covidresearchtrials.com

Author:
Shray Alag

Method Summary

All Methods | Static Methods | Instance Methods | Concrete Methods

Modifier and Type	Method and Description
java.util.List<java.lang.String>	getClinicalTrialIds()<br>Get the List of relevant clinical trial ids
java.util.Collection<java.lang.String>	getClinicalTrialsForDrugTerm(java.lang.String drugId)<br>Get the list of relevant clinical trial ids for the given drug id
java.util.List<java.lang.String>	getDrugIds()<br>Returns a List of all unique drug ids
java.lang.String	getDrugName(java.lang.String id)<br>Get the drug name for the given drug id
java.util.Collection<java.lang.String>	getDrugTermsForClinicalTrial(java.lang.String clinicalTrialId)<br>Get the list of drug terms for a clinical trial using clinical trial id
static DrugToClinicalTrials	getInstance()<br>Public static getInstance method
static void	main(java.lang.String[] args)<br>Sample program

Methods inherited from class java.lang.Object

equals, getClass, hashCode, notify, notifyAll, toString, wait, wait, wait

Method Detail

getInstance

```
public static DrugToClinicalTrials getInstance()
```

Public static getInstance method

Returns:
DrugToClinicalTrials instance

getDrugIds

```
public java.util.List<java.lang.String> getDrugIds()
```

Returns a List of all unique drug ids

Returns:
Drug ids that can be used to get drug names

getDrugName

```
public java.lang.String getDrugName(java.lang.String id)
```

Get the drug name for the given drug id

Figure 2. Screenshot of the API page for accessing the list of COVID-19 drugs/interventions (http://covidresearchtrials.com/08162020/api/doc/index.html). Class: com.covidresearchtrials.ct.drugs.DrugToClinicalTrials

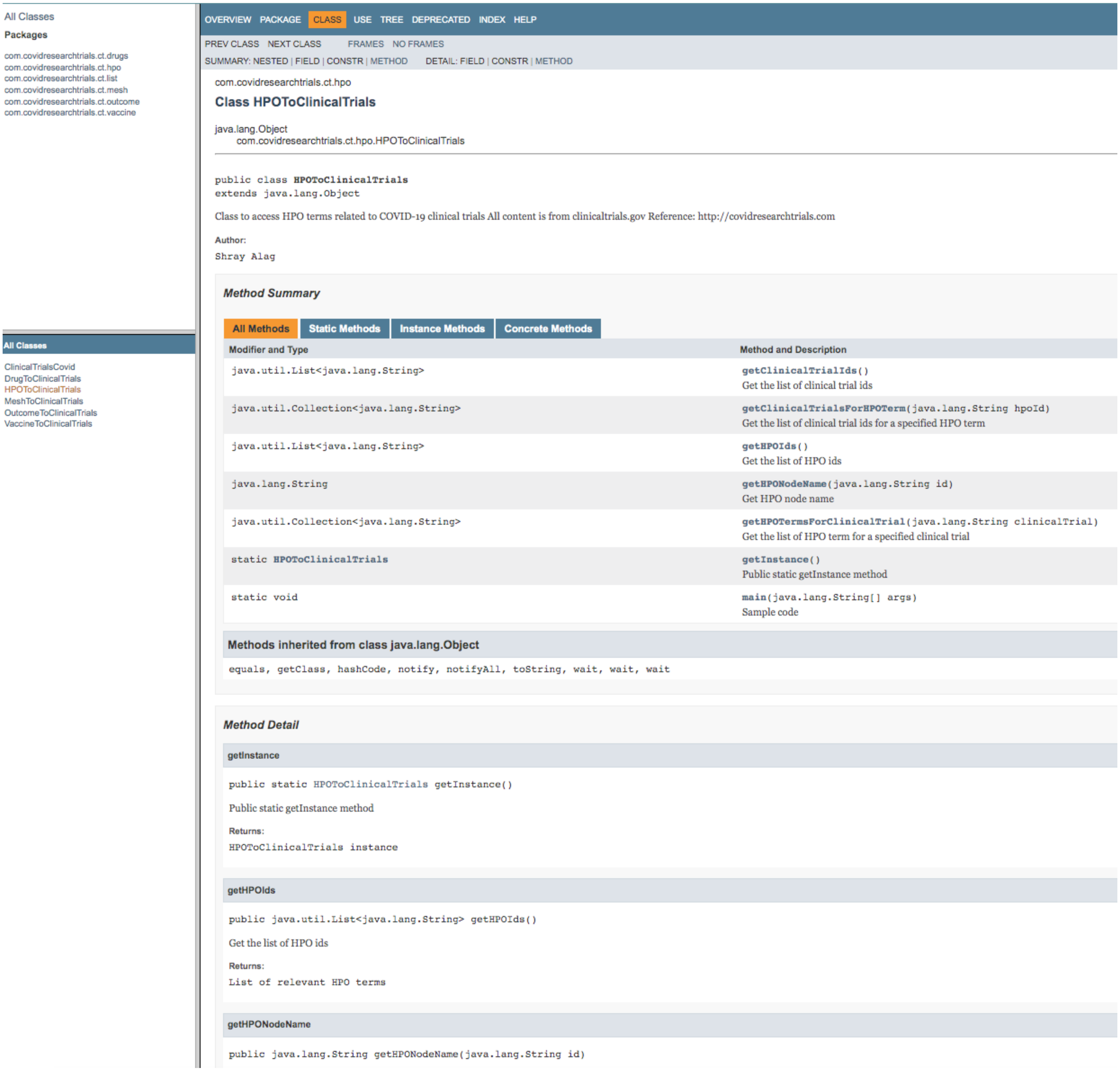

Figure 3. Screenshot of the API page for accessing the list of HPO nodes related to COVID-19 clinical trials (http://covidresearchtrials.com/08162020/api/doc/index.html). Class: com.covidresearchtrials.ct.hpo.HPOToClinicalTrials

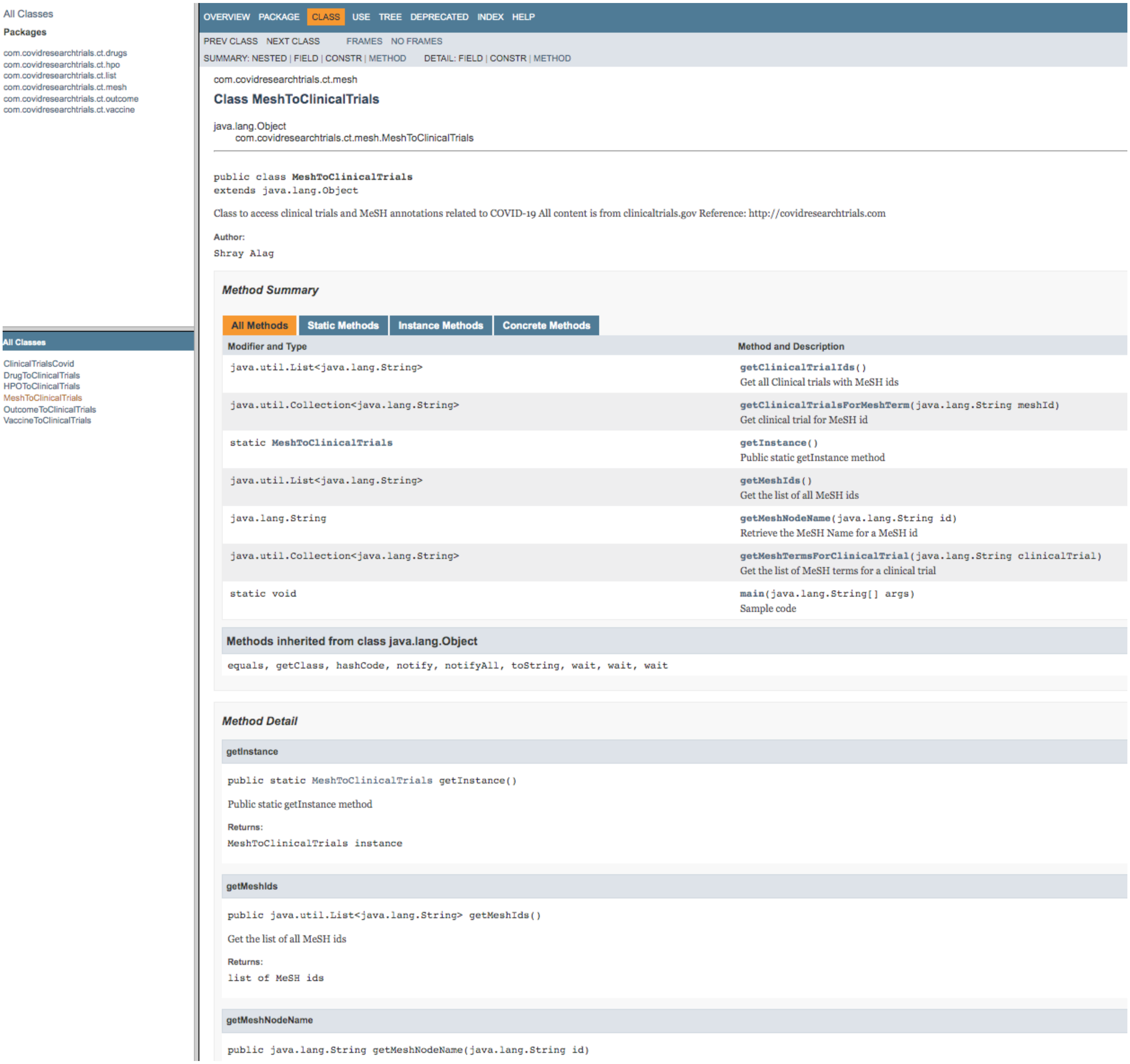

All Classes

Packages

com.covidresearchtrials.ct.drugs
com.covidresearchtrials.ct.hpo
com.covidresearchtrials.ct.list
com.covidresearchtrials.ct.mesh
com.covidresearchtrials.ct.outcome
com.covidresearchtrials.ct.vaccine

All Classes

ClinicalTrialsCovid
DrugToClinicalTrials
HPOToClinicalTrials
MeshToClinicalTrials
OutcomeToClinicalTrials
VaccineToClinicalTrials

OVERVIEW PACKAGE CLASS USE TREE DEPRECATED INDEX HELP

PREV CLASS NEXT CLASS FRAMES NO FRAMES

SUMMARY: NESTED | FIELD | CONSTR | METHOD DETAIL: FIELD | CONSTR | METHOD

com.covidresearchtrials.ct.mesh

Class MeshToClinicalTrials

java.lang.Object
    com.covidresearchtrials.ct.mesh.MeshToClinicalTrials

```
public class MeshToClinicalTrials
extends java.lang.Object
```

Class to access clinical trials and MeSH annotations related to COVID-19 All content is from clinicaltrials.gov Reference: http://covidresearchtrials.com

Author:

Shray Alag

Method Summary

All Methods | Static Methods | Instance Methods | Concrete Methods

Modifier and Type	Method and Description
`java.util.List<java.lang.String>`	`getClinicalTrialIds()`<br>Get all Clinical trials with MeSH ids
`java.util.Collection<java.lang.String>`	`getClinicalTrialsForMeshTerm(java.lang.String meshId)`<br>Get clinical trial for MeSH id
`static MeshToClinicalTrials`	`getInstance()`<br>Public static getInstance method
`java.util.List<java.lang.String>`	`getMeshIds()`<br>Get the list of all MeSH ids
`java.lang.String`	`getMeshNodeName(java.lang.String id)`<br>Retrieve the MeSH Name for a MeSH id
`java.util.Collection<java.lang.String>`	`getMeshTermsForClinicalTrial(java.lang.String clinicalTrial)`<br>Get the list of MeSH terms for a clinical trial
`static void`	`main(java.lang.String[] args)`<br>Sample code

Methods inherited from class java.lang.Object

`equals, getClass, hashCode, notify, notifyAll, toString, wait, wait, wait`

Method Detail

getInstance

```
public static MeshToClinicalTrials getInstance()
```

Public static getInstance method

Returns:

`MeshToClinicalTrials instance`

getMeshIds

```
public java.util.List<java.lang.String> getMeshIds()
```

Get the list of all MeSH ids

Returns:

`list of MeSH ids`

getMeshNodeName

```
public java.lang.String getMeshNodeName(java.lang.String id)
```

Figure 4. Screenshot of the API page for accessing the list of MeSH nodes related to COVID-19 clinical trials (http://covidresearchtrials.com/08162020/api/doc/index.html). Class: com.covidresearchtrials.ct.mesh.MeshToClinicalTrials

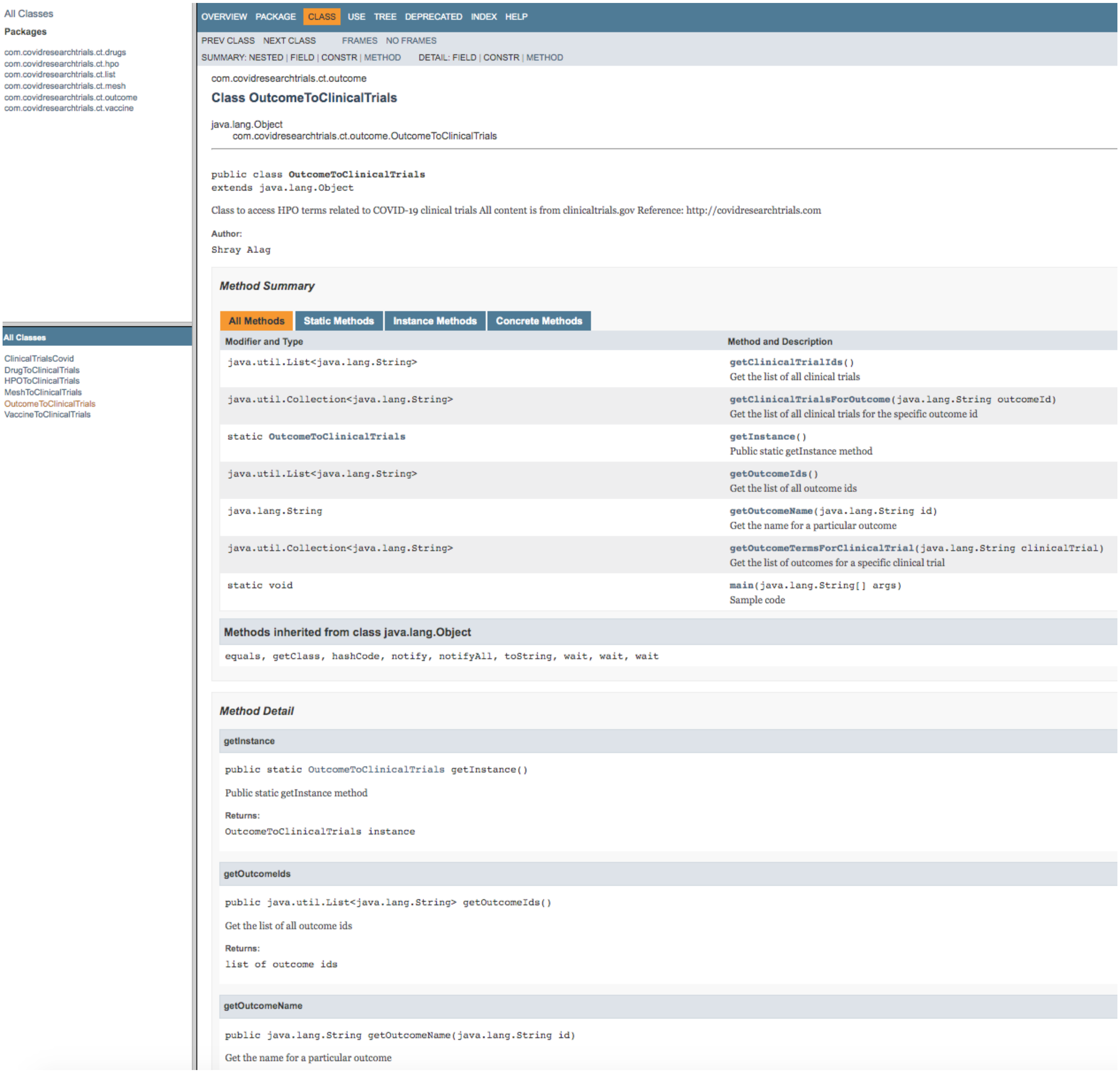

All Classes

Packages

com.covidresearchtrials.ct.drugs
com.covidresearchtrials.ct.hpo
com.covidresearchtrials.ct.list
com.covidresearchtrials.ct.mesh
com.covidresearchtrials.ct.outcome
com.covidresearchtrials.ct.vaccine

All Classes

ClinicalTrialsCovid
DrugToClinicalTrials
HPOToClinicalTrials
MeshToClinicalTrials
OutcomeToClinicalTrials
VaccineToClinicalTrials

OVERVIEW PACKAGE CLASS USE TREE DEPRECATED INDEX HELP

PREV CLASS NEXT CLASS FRAMES NO FRAMES

SUMMARY: NESTED | FIELD | CONSTR | METHOD DETAIL: FIELD | CONSTR | METHOD

com.covidresearchtrials.ct.outcome

Class OutcomeToClinicalTrials

java.lang.Object
com.covidresearchtrials.ct.outcome.OutcomeToClinicalTrials

```
public class OutcomeToClinicalTrials
extends java.lang.Object
```

Class to access HPO terms related to COVID-19 clinical trials All content is from clinicaltrials.gov Reference: http://covidresearchtrials.com

Author:
Shray Alag

Method Summary

All Methods | Static Methods | Instance Methods | Concrete Methods

Modifier and Type	Method and Description
java.util.List<java.lang.String>	**getClinicalTrialIds**() Get the list of all clinical trials
java.util.Collection<java.lang.String>	**getClinicalTrialsForOutcome**(java.lang.String outcomeId) Get the list of all clinical trials for the specific outcome id
static OutcomeToClinicalTrials	**getInstance**() Public static getInstance method
java.util.List<java.lang.String>	**getOutcomeIds**() Get the list of all outcome ids
java.lang.String	**getOutcomeName**(java.lang.String id) Get the name for a particular outcome
java.util.Collection<java.lang.String>	**getOutcomeTermsForClinicalTrial**(java.lang.String clinicalTrial) Get the list of outcomes for a specific clinical trial
static void	**main**(java.lang.String[] args) Sample code

Methods inherited from class java.lang.Object

equals, getClass, hashCode, notify, notifyAll, toString, wait, wait, wait

Method Detail

getInstance

```
public static OutcomeToClinicalTrials getInstance()
```

Public static getInstance method

Returns:
OutcomeToClinicalTrials instance

getOutcomeIds

```
public java.util.List<java.lang.String> getOutcomeIds()
```

Get the list of all outcome ids

Returns:
list of outcome ids

getOutcomeName

```
public java.lang.String getOutcomeName(java.lang.String id)
```

Get the name for a particular outcome

Figure 5. Screenshot of the API page for accessing the list of Outcomes related to COVID-19 clinical trials (http://covidresearchtrials.com/08162020/api/doc/index.html). Class: com.covidresearchtrials.ct.outcome.OutcomeToClinicalTrials

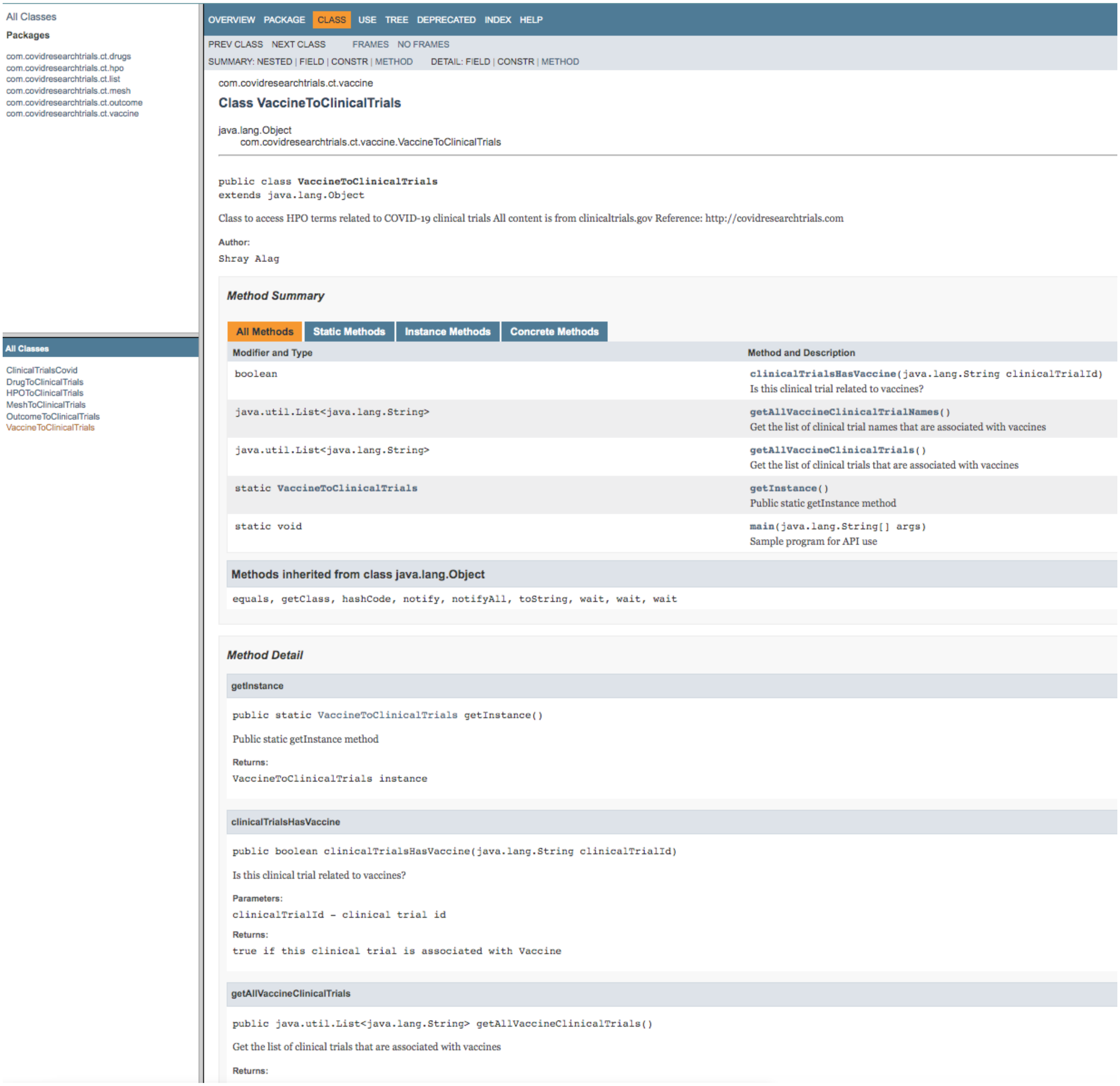

Figure 6. Screenshot of the API page for accessing the list of COVID-19 vaccine clinical trials (http://covidresearchtrials.com/08162020/api/doc/index.html). Class: com.covidresearchtrials.ct.vaccine.VaccineToClinicalTrials
